# Prevalence of bovine viral diarrhea virus in cattle between 2010 and 2021: A global systematic review and meta-analysis

**DOI:** 10.3389/fvets.2022.1086180

**Published:** 2023-01-17

**Authors:** Nuo Su, Qi Wang, Hong-Ying Liu, Lian-Min Li, Tian Tian, Ji-Ying Yin, Wei Zheng, Qing-Xia Ma, Ting-Ting Wang, Ting Li, Tie-Lin Yang, Jian-Ming Li, Nai-Chao Diao, Kun Shi, Rui Du

**Affiliations:** ^1^College of Chinese Medicine Materials, Jilin Agricultural University, Changchun, China; ^2^College of Animal Science and Technology, Jilin Agricultural University, Changchun, China; ^3^Laboratory of Production and Product Application of Sika Deer of Jilin Province, Jilin Agricultural University, Changchun, China; ^4^Key Laboratory of Animal Production, Product Quality and Security, Ministry of Education, Jilin Agricultural University, Changchun, China

**Keywords:** bovine viral diarrhea virus, cattle, meta-analysis, antigen prevalence, antibody prevalence, risk factors

## Abstract

**Background:**

Bovine viral diarrhea is one of the diseases that cause huge economic losses in animal husbandry. Many countries or regions have successively introduced eradication plans, but BVDV still has a high prevalence in the world. This meta-analysis aims to investigate the prevalence and risk factors of BVDV in the world in recent 10 years, and is expected to provide some reference and theoretical basis for BVDV control plans in different regions.

**Method:**

Relevant articles published from 2010 to 2021 were mainly retrieved from NCBI, ScienceDirect, Chongqing VIP, Chinese web of knowledge (CNKI), web of science and Wanfang databases.

**Results:**

128 data were used to analyze the prevalence of BVDV from 2010 to 2021. BVDV antigen prevalence rate is 15.74% (95% CI: 11.35–20.68), antibody prevalence rate is 42.77% (95% CI: 37.01–48.63). In the two databases of antigen and antibody, regions, sampling time, samples, detection methods, species, health status, age, sex, breeding mode, and seasonal subgroups were discussed and analyzed, respectively. In the antigen database, the prevalence of dairy cows in the breed subgroup, ELISA in the detection method subgroup, ear tissue in the sample subgroup, and extensive breeding in the breeding mode were the lowest, with significant differences. In the antibody database, the prevalence rate of dairy cows in the breed subgroup and intensive farming was the highest, with a significant difference. The subgroups in the remaining two databases were not significantly different.

**Conclusion:**

This meta-analysis determined the prevalence of BVDV in global cattle herds from 2010 to 2021. The prevalence of BVDV varies from region to region, and the situation is still not optimistic. In daily feeding, we should pay attention to the rigorous and comprehensive management to minimize the spread of virus. The government should enforce BVDV prevention and control, implement control or eradication policies according to local conditions, and adjust the policies in time.

## 1. Introduction

Bovine viral diarrhea virus (BVDV) is the main pathogen of bovine viral diarrhea BVD (1, 2), and it is the main member of flaviviridae and pestivirus genus, which consists of three species: pestivirus A (BVDV-1), pestivirus B (BVDV-2) and pestivirus H (bovine viral diarrhea virus type 3 [Hobi-like pestiviruses)] (WOAH). BVDV-1 contains at least 22 subgenotypes of 1a-1v and BVDV-2 and HoBi-like pestivirus are divided into 4 subtypes (3). Multiple species and genotypes lead to the mutation of BVDV, which brings great obstacles to its prevention and control. BVDV contains two biotypes, and BVDV can be divided into cytopathic type (CP) and non-cytopathic type (NCP) according to whether it causes pathological changes in cultured tissue cells (4). NCP BVDV can infect cows early in embryonic development and produce persistently infected (PI) calves. PI makes it more difficult to control BVDV. It is the main source of infection of BVDV, because it is immune tolerant to infected strains, does not produce antibodies, and is always infected and continuously detoxifies (5). In contrast, the risk of transient infection (TI) transmission is weaker, producing only mild clinical symptoms to the host and expelling the virus into the environment for a short period of time. However, TI damage to the immune system can exacerbate the occurrence of secondary infections, so it remains an important component of BVDV infection (6).

BVDV is widespread in the world and can cause gastrointestinal, respiratory and reproductive diseases. The induced immunosuppression can increase the probability of infection of other diseases (7). BVDV reduces the breeding and growth efficiency of livestock through various ways, increases the mortality rate of young animals and the prevalence rate of reproductive system, respiratory system and gastrointestinal diseases, and causes continuous and serious economic losses to the animal husbandry (8). BVDV can infect cattle, goats, sheep, camels, pigs and other cloven-hoofed animals (9–11). Among them, cattle are the main infection host and source of BVDV, and are most affected by diseases (12). As a major economic animal, cattle are closely related to people's life. According to the survey, the economic impact of BVD ranges from £0 to £552 per cow per year, with a mean impact of £46.50 (13). At the same time, BVDV's pollution to bovine-derived substances further endangers the accuracy of scientific research and the safety of biological products such as vaccines (14). The growing demand for beef and dairy products reminds people to focus on the health of primitive animals and avoid possible economic losses (15). Therefore, it is very important to investigate and control the prevalence of BVDV infection in cattle species. ACVIM's consensus statement clarifies the importance of BVDV control (16). Many countries have also introduced measures to control and purify BVDV. Denmark introduced the BVD eradication plan as early as 1994 (17). Northern Ireland began implementing the BVD AHWNI eradication program in 2013 and the virus positivity declined significantly by 2020 (18). Germany's 6-year mandatory plan has seen a significant decline in the number of PI by 2016, and further removal of the virus is the next challenge (19). Switzerland has had a control program since 2008 and infection rates have dropped significantly by 2020, but PI animals remain the last strong obstacle (20). In 2016–2017, the Indonesian government tried to breed beef cattle by increasing artificial insemination, hoping to reduce the vertical transmission of BVDV (21).

According to the positive rate of BVDV in different species, many articles have been meta-analyzed. Knowing the prevalence of BVDV in time can not only provide data support for the formulation of BVDV prevention and control policies, but also provide technical guidance for practical production.

This paper makes a meta-analysis on the prevalence of BVDV infection among cattle in the world in recent 10 years. Through the summary of the latest data and the thinking caused by eradication plans in different regions, the following questions are addressed: “What should we do to control BVDV? How should the control plan be carried out under different circumstances?”. We hope to observe the effectiveness of current prevention and control measures and provide reference for further prevention and control of BVDV in the future.

## 2. Materials and methods

### 2.1. Search strategy

We searched six databases of PubMed, ScienceDirect, Web of Science, CNKI, VIP, and Wanfang, and find articles published in Chinese and English from 2010 to May 20, 2021. Designed to filter prevalence data for all BVDV, the specific search process is as follows:

PubMed search strategy is as follows: According to MeSH terminology, the following keywords were used to search: “Diarrhea Viruses, Bovine Viral,” “Cattle,” and the Boolean operators “OR,” “AND” in the “Keyword/Title/Summary” field alone or in combination.

A: We search for “Cattle” based on MeSH terminology: ((((((((((((((((((((“Cattle”[Mesh]) OR (Cow)) OR (Cows)) OR (Bos indicus)) OR (Zebu)) OR (Zebus)) OR (Holstein Cow)) OR (Cow, Holstein)) OR (Dairy Cow)) OR (Cow, Dairy)) OR (Dairy Cows)) OR (Beef Cow)) OR (Beef Cows)) OR (Cow, Beef)) OR (Bos grunniens)) OR (Yak)) OR (Yaks)) OR (Bos taurus)) OR (Cow, Domestic)) OR (Domestic Cow)) OR (Domestic Cows).

B: We search for “Diarrhea Viruses, Bovine Viral” based on MeSH terminology: (((((((((((“Diarrhea Viruses, Bovine Viral”[Mesh]) OR (Bovine Viral Diarrhea Viruses)) OR (Bovine Pestivirus)) OR (Bovine Pestiviruses)) OR (Pestiviruses, Bovine)) OR (Bovine Diarrhea Virus)) OR (Bovine Diarrhea Viruses)) OR (Diarrhea Virus, Bovine)) OR (Diarrhea Viruses, Bovine)) OR (Virus, Bovine Diarrhea)) OR (Viruses, Bovine Diarrhea)) OR (Diarrhea Virus, Bovine Viral).

C: We used the Boolean operators “OR” for the entry terms and “AND” for the MeSH terms. (((((((((((((((((((((“Cattle”[Mesh]) OR (Cow)) OR (Cows)) OR (Bos indicus)) OR (Zebu)) OR (Zebus)) OR (Holstein Cow)) OR (Cow, Holstein)) OR (Dairy Cow)) OR (Cow, Dairy)) OR (Dairy Cows)) OR (Beef Cow)) OR (Beef Cows)) OR (Cow, Beef)) OR (Bos grunniens)) OR (Yak)) OR (Yaks)) OR (Bos taurus)) OR (Cow, Domestic)) OR (Domestic Cow)) OR (Domestic Cows)) AND ((((((((((((“Diarrhea Viruses, Bovine Viral”[Mesh]) OR (Bovine Viral Diarrhea Viruses)) OR (Bovine Pestivirus)) OR (Bovine Pestiviruses)) OR (Pestiviruses, Bovine)) OR (Bovine Diarrhea Virus)) OR (Bovine Diarrhea Viruses)) OR (Diarrhea Virus, Bovine)) OR (Diarrhea Viruses, Bovine)) OR (Virus, Bovine Diarrhea)) OR (Viruses, Bovine Diarrhea)) OR (Diarrhea Virus, Bovine Viral)).

Use advanced search in ScienceDirect and Web of Science databases to improve the accuracy of your results, enter subject terms “cattle,” “Diarrhea Viruses, Bovine Viral,” “prevalence” and select research articles to search. The VIP database was searched for articles by selecting the subject headings “bovine” and “bovine viral diarrheal mucosal disease” or “bovine” and “bovine viral diarrhea virus.” Wanfang and CNKI search strategies are: The theme words “bovine” and “bovine viral diarrhea mucosal disease” or: “bovine” and “bovine viral diarrhea virus” or “bovine” and “BVDV” were selected.

In order to collect comprehensive data as much as possible, Google Academic will further search the related articles of the collected articles.

### 2.2. Inclusion criteria and exclusion criteria

Eligible articles are screened according to the inclusion exclusion criteria below.

Inclusion criteria:

(1) Study on the prevalence of BVDV infection;
(2) Literature between 2010 and 2021.5.20;
(3) The species is cattle and the source is clear;
(4) The type of article is experimental research article;
(5) Literature published in Chinese or English.

Exclusion criteria:

(1) Repetitive articles;
(2) Articles that cannot be downloaded;
(3) Study animals were vaccinated or model animals;
(4) Research data is not clear;
(5) Sample size <30.

### 2.3. Data extraction

Import the search database results into the EndNote (EndNote X 9.3.3) reference manager software (Clarivate analysis, Philadelphia, Pennsylvania, USA) for screening, delete duplicate articles, and then two reviewers further screen according to the article title and abstract. Obtain key data information from all relevant studies, including the first author, sampling year, country, mainland, sample type, detection method, variety, season, health status, age, gender and breeding mode. Microsoft ^®^ Excel ^®^ 2019 MSO (16.0.14228.20216) 32 is used to sort and compile the data mentioned above.

### 2.4. Quality assessment

The level of proposal evaluation, formulation and evaluation methods determines the quality of selected literature. The scoring standard includes the following four aspects, whether it is random sampling, sampling time, whether the sampling method is detailed, whether the detection method is detailed, and whether there are more than four factors. “Yes” is 1 point, and the maximum is 5 points. Based on the above standards, the article is divided into three grades 0–1, 2–3, 4–5, respectively.

### 2.5. Statistical analysis

Under the guidance of PRISMA 2020, the article strictly follows its requirements and completes the systematic evaluation and meta-analysis (Supplementary material 1).

R software 4.0.0 is used to compile and calculate data. Sensitivity analyses were performed in different possible ways for all included studies, and bias tests were done by looking at funnel graphs (22). Egger's test and trim and fill analysis further illustrate whether bias occurs (23). Bias is indicated when the funnel chart is asymmetrical or when the Egger test *p* < 0.05. Q- test (X^2^ and p representation) was used to evaluate the heterogeneity among the studies, and the forest map was used for visual analysis. The degree of heterogeneity was further evaluated by I^2^ (24). The higher the I^2^, the greater the heterogeneity. The code in R for meta-analysis is in Supplementary material 2. Factors investigated in the subgroup analysis included sampling year (before 2017, after 2017), season, health status (healthy, clinically symptomatic), age (<6 months, >6 months), country, region, test method, sample origin, breed (beef cattle, dairy cows, dairy meat dual-use, breeding cattle), sex, breeding pattern (intensive, extensive).

## 3. Results

### 3.1. Flow chart and results of literature screening

**Figure 1:**
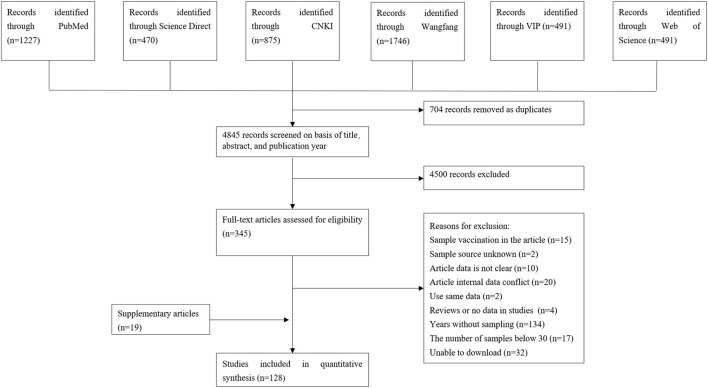
Flow diagram of eligible studies for searching and selecting.

A total of 5,549 eligible articles were obtained. Seven hundred four duplicate articles were deleted, and 4,500 articles were further screened according to the title, abstract, and Year of publication. Further screening according to the inclusion and exclusion criteria, 15 articles on vaccination were deleted, 2 sample sources were unclear, 10 article data were unclear, 20 data errors were used, 2 articles were used the same data, 4 non-epidemiological investigative articles, 134 non-sampling years, 17 articles with a sample size of <30, 32 articles that could not be queried, and a total of 109 articles were included. Nineteen articles were added to Google Academic, including 128 articles in total. The specific flow chart is shown in Figure 1.

### 3.2. Studies included

Through literature screening, 128 studies were eligible for the meta-analysis. Among them, there were 77 articles on detecting antibodies and 51 articles on detecting antigens. Studies were identified from 19 countries worldwide, including 10 countries in Asia, two in North America, two in South America, two in Europe and three in Africa (Supplementary Figure 1).

There are a total of 51 antigen detection articles, including 27 articles of 4–5 points and 24 articles of 2–3 points (Table 1). A total of 46,211 cattle were tested, and 3,488 BVDV-positive cattle were tested, with a positive infection rate of 15.74% (95% CI: 11.35–20.68 3,488/46,211, Table 2). Among the regional subgroups, Europe had the highest positive rate with a positive rate of 23.27% (95% CI: 0.00–89.41, Table 2), followed by the Asia positivity rate of 16.75% (95% CI: 11.27–23.04, Table 2), The lowest is 0.32% (95% CI: 0.20–0.46, Table 2) in North America. Spain (59.40%, 95% CI: 50.91–67.62, Supplementary Table 1) has the highest antigen-positive rate among all countries and India has the lowest positive rate. The positivity rate after 2017 was higher than before 2017. The positive rate of ear tissue in the test samples was the lowest, with a positive rate of 0.48% (95% CI: 0.05–1.20, Table 2), which was significantly different. Diary cows had the lowest positive rate of infection, with a positive rate of 11.43% (95% CI: 6.61–17.32, Table 2), which was significantly different. In the health condition subgroup, the rate of BVDV infection with clinical symptoms was higher than that of clinically healthy cattle. Summer infection rate was lowest, with a positive rate of 4.17% (95% CI: 0.12–12.59, Table 2), Spring positivity rate was highest at 21.33% (95% CI: 0.82–57.99, Table 2). ELISA had the lowest positive rate among the test methods, with a positive rate of 6.94% (95% CI: 2.12–14.16, Table 2), which was significantly different. The positive rate in adult cattle is higher than that in calves. Extensive culture mode had the lowest rate of infection, with a positive rate of 1.11% (95% CI: 0.00–5.86, Table 2), which was significantly different.

**Table 1:** Included studies of Bovine viral diarrhea virus infection of cattle in the word.

**Reference ID**	**Country**	**Sampling time**	**Detection method**	**No. tested**	**No. positive**	**Prevalence**	**Study design**	Score^*^
**Asia**								
Xu et al. (25)	China	2018–2019	PCR^*^	232	26	0.112068966	Cross sectional	3
Deng et al. (26)	China	2017	PCR	901	20	0.022197558	Cross sectional	3
Guo et al. (27)	China	2018.3–2019.5	PCR	302	135	0.447019868	Cross sectional	3
Chang et al. (28)	China	2019	PCR	1,234	89	0.072123177	Cross sectional	3
Zhang et al. (29)	China	2017–2018	PCR	535	185	0.345794393	Cross sectional	4
Long (30)	China	2017, 2018	PCR	76	21	0.276315789	Cross sectional	4
Wang et al. (31)	China	2011.09-2012.03	ELISA^*^	1,434	23	0.016039052	Cross sectional	5
Lee et al. (32)	Korea	2014.07–2016.06	PCR	207	14	0.067632850	Cross sectional	3
Li (33)	China	2018.2.24–2019.2.27	ELISA	305	17	0.055737705	Cross sectional	4
Wang (34)	China	2015.7–2016.11	Colloidal gold	232	35	0.150862069	Cross sectional	4
Wang et al. (35)	China	2015.6–7	PCR	81	6	0.074074074	Cross sectional	3
Chen et al. (36)	China	2016	PCR	149	29	0.194630872	Cross sectional	3
Li (37)	China	2017–2019	PCR	109	87	0.798165138	Cross sectional	4
Yan et al. (38)	China	2017	PCR	138	62	0.449275362	Cross sectional	4
Yang et al. (38)	China	2017	PCR	74	28	0.378378378	Cross sectional	5
Sun and Qin (39)	China	2017–2018	ELISA	114	28	0.245614035	Cross sectional	4
Han et al. (40)	Korea	2016	PCR	143	87	0.608391608	Cross sectional	4
Ryu and Choi (41)	Korean	2017.3–2018.10	PCR	635	35	0.055118110	Cross sectional	5
Kim et al. (42)	Korean	2013	ELISA	3,050	21	0.006885246	Cross sectional	4
Zhang (43)	China	2014.1–2016.1	PCR	173	24	0.138728324	Cross sectional	3
Luo et al. (44)	China	2014.3–2014.12	PCR	248	28	0.112903226	Cross sectional	3
Quan and Liu (45)	China	2012.3–2013.6	PCR	184	27	0.146739130	Cross sectional	3
Lv and Zhang (46)	China	2013.1–4	PCR	252	58	0.230158730	Cross sectional	3
Liu et al. (47)	China	2016	ELISA	346	317	0.916184971	Cross sectional	5
Kaveh et al. (48)	Iran	2015.7–12	PCR	128	26	0.203125000	Cross sectional	3
Wang et al. (49)	China	2017	ELISA	1,160	14	0.012100000	Cross sectional	4
Wang (50)	China	2019	PCR	200	35	0.175000000	Cross sectional	4
Li et al. (51)	China	2011–2012	Bio-X detection kit	80	71	0.888000000	Cross sectional	3
Agah et al. (52)	Japan	2015.12–2016.9	ELISA	1,075	2	0.001860465	Cross sectional	4
Zhang et al. (53)	China	2018.7	PCR	1,286	4	0.003110420	Cross sectional	4
Yao et al. (54)	China	2016–2017	PCR	145	19	0.131034483	Cross sectional	3
Song et al. (55)	China	2016–2017	PCR	382	101	0.264397906	Cross sectional	3
Wang and Man (56)	China	2017, 2018, 2019	PCR	690	114	0.165217391	Cross sectional	3
Wei et al. (57)	China	2019	PCR	640	256	0.400000000	Cross sectional	3
Dehkordi (58)	Iran	2010	PCR	992	203	0.204637097	Cross sectional	3
Lv et al. (58)	China	2011.5–2011.9	ELISA	464	2	0.004310345	Cross sectional	5
Weng (59)	China	2010–2013	PCR	4,327	18	0.004159926	Cross sectional	3
Zhang et al. (60)	China	2014.11	ELISA	920	34	0.036956522	Cross sectional	5
Mishra et al. (61)	India	2012–2013	PCR	1,049	1	0.000953289	Cross sectional	5
Gangil et al. (62)	India	2016.9–2018.3	ELISA	55	0	0	Cross sectional	3
Alam et al. (63)	Bangladesh	2015.1–2015.12	UN^*^	644	21	0.032608696	Cross sectional	2
Yitagesu et al. (64)	Bangladesh	2018.9–2019.10	ELISA	882	0	0	Cross sectional	5
Asmare et al. (65)	Bangladesh	2012	ELISA	563	185	0.328596803	Cross sectional	4
**Europe**								
Fernández-Aguilar et al. (66)	Spain	2010, 2011, 2012	PCR	133	79	0.593984962	Cross sectional	3
Decaro et al. (67)	Italy	2015–2016	PCR	1,005	17	0.016915423	Cross sectional	3
**South America**								
Caffarena et al. (68)	Uruguay	2016.1–11	PCR	480	6	0.012500000	Cross sectional	4
Maya et al. (69)	Uruguay	2015.3–2017.12	PCR	2546	23	0.009033778	Cross sectional	3
Paixão et al. (70)	Brazil	2014.08–2014.12	VN^*^	305	110	0.360655738	Cross sectional	4
Viana et al. (71)	Brazil	2013.3–5	VN	400	157	0.392500000	Cross sectional	4
Freitas et al. (72)	Brazil	2015.5–2018.8	ELISA	6465	115	0.017788090	Cross sectional	4
**North America**								
Stephenson et al. (73)	United States	2005.3–12	IHC	7,544	24	0.003181336	Cross sectional	5

UN^*^, Unclear; PCR^*^, Polymerase chain reaction; ELISA^*^, Enzyme linked immunosorbent assay; VN^*^, Virus neutralization; Score^*^, quality assessment.

**Table 2:** Antigen prevalence of Bovine viral diarrhea virus of cattle in the word.

	**No. studies**	**No. tested**	**No. positive**	% (95% CI^*^)	**Heterogeneity**			**Univariate meta-regression**	
					**χ^2^**	***P*-value**	**I^2^ (%)**	***P*-value**	**Coefficient (95% CI)**
**Area** ^*^									
Asia	43	27,333	2,957	16.75% (11.27–23.04)	7,017.61	0.00	99.4%	0.3463	0.0839 (−0.0907–0.2585)
Europe	2	1,138	96	23.27% (0.00–89.41)	263.03	<0.01	99.6%	
North America	1	7,544	24	0.32% (0.20–0.46)	0.00	–	–	
South America	5	10,196	411	10.55% (2.63–22.82)	778.30	<0.01	99.5%	
**Sampling years**									
Before 2017	30	26,608	1,625	17.18% (11.08–24.27)	5,317.36	0.00	99.5%	
After 2017	21	9,513	1,156	17.91% (10.33–26.99)	2,204.72	0.00	99.1%	0.8994	0.0088 (−0.1281–0.1457)
**Sample**									
Blood sample	24	17,065	1,644	17.15% (9.50–26.47)	4,850.39	0.00	99.5%	
Fecal sample	15	3,568	751	20.89% (12.97–30.08)	529.49	<0.01	97.4%	
Ear tissue	7	20,159	184	0.48% (0.05–1.20)	120.65	<0.01	95.0%	<0.0001	−0.3602 (−0.5326– −0.1878)
Others	9	1,689	413	17.40% (8.16–28.99)	163.55	<0.01	95.1%	
**Breed**									
Diary	18	15,819	844	11.43% (6.61–17.32)	1,626.27	0.00	99.0%	0.0213	−0.1835 (−0.3397– −0.0273)
Beef	8	2,679	688	23.60% (3.74–53.08)	1,681.94	0.00	99.6%	
Both dairy and beef	9	1,386	391	27.50% (15.68–41.14)	218.56	<0.01	96.3%	
**Health condition**									
Clinical symptoms	25	12,557	1,326	22.56% (13.69–32.86)	3,032.63	0.00	99.2%	
Healthy	11	3,758	582	13.72% (5.15–25.46)	825.77	<0.01	98.8%	0.2559	−0.1156 (−0.3150– 0.0838)
**Season** ^*^									
Spring	2	950	200	21.33% (0.82–57.99)	142.88	<0.01	99.3%	
Summer	4	1,854	46	4.17% (0.12–12.59)	87.13	<0.01	96.6%	0.2166	−0.1454 (−0.3759– 0.0852)
Autumn	3	2,031	243	8.64% (0.71–23.60)	149.23	<0.01	98.7%	
Winter	3	767	123	10.41% (0.00–37.25)	162.78	<0.01	98.8%	
**Detection method** ^*^									
PCR	32	19,676	1,860	16.97% (11.23–23.60)	3,972.00	0.00	99.2%	
ELISA	13	16,833	758	6.94% (2.12–14.16)	2,499.08	0.00	99.5%	0.0164	−0.1797 (−0.3265– −0.0329)
VN	2	705	267	37.87% (34.31–41.48)	0.74	0.39	0.0%	
Others	3	7,856	130	27.82% (0.00–78.56)	531.22	<0.01	99.6%	
**Age**									
Calf	15	11,552	565	15.03% (6.56–26.09)	1,518.17	<0.01	99.1%	
Adult cattle	6	1,689	318	23.42% (5.93–47.09)	358.91	<0.01	98.6%	0.3176	0.1250 (−0.1202–0.3702)
**Breeding mode**									
Intensive	28	29,750	1,912	15.07% (9.37–21.82)	5,841.32	0.00	99.5%	
Extensive	3	1,981	37	1.11% (0.00–5.86)	74.53	<0.01	97.3%	0.0255	−0.2901 (−0.5447– −0.0356)
Mixed culture	5	8,945	144	14.81% (4.01–30.62)	448.57	<0.01	99.1%	
Total		51	46,211	3,488	15.74% (11.35–20.68)	9,132.89	0.00	99.5%	

CI^*^: Confidence interval.

Area^*^: Africa: Egypt, Ethiopia, Morocco; Asia: China, India; Europe: Italy, Spain, Portugal, France, Poland; North America: USA; Oceania: Australia; South America: Brazilian.

Method^*^: ELISA: Enzyme-linked immunosorbent assay; PCR: Polymerase chain reaction; VN: virus neutralization.

Season^*^: Spring: Mar. to May.;Summer: Jun. to Aug.;Autumn: Sep. to Nov.;Winter: Dec. to Feb.

A total of 77 articles were published on the detection of BVDV antibodies, including 43 articles with 4–5 points and 34 articles with 2–3 points (Table 3). A total of 55,349 samples were tested, of which 24,585 were positive, and the positive rate was 42.77% (95% CI: 37.01–48.63, Table 4). South America had the highest prevalence in the regional subgroup, with a positivity rate of 76.4% (95% CI: 72.06–80.50, Table 4, Supplementary Table 2) followed by North America, Africa, Europe, and Asia. Infection rates have decreased after 2017 compared to before 2017. Dairy cattle had the highest prevalence rate, with a positive rate of 48.68% (95% CI: 39.19–58.22, Table 4), which was significantly different. In the health condition subgroup, the infection rate of clinically healthy cattle was relatively low, with a positive rate of 43.80% (95% CI: 26.57–61.83, Table 4). The positive rate is relatively high in summer 60.16% (95% CI: 48.92–70.89, Table 4) and winter 63.44% (95% CI: 35.15–87.45, Table 4). The positive rate for cows is lower than that of bulls, and the positive rate of calves is lower than that of adult cattle. Intensive has the highest prevalence of all culture models, with a positive rate of 50.35% (95% CI: 42.93–57.76, Table 4).

**Table 3:** Included studies of Bovine viral diarrhea virus infection of cattle in the word.

**Reference ID**	**Country**	**Sampling time**	**Detection method**	**No. tested**	**No. positive**	**Prevalence**	**Study design**	Score^*^
**Africa**								
Demil et al. (74)	Ethiopia	2017.12–2018.7	ELISA	339	91	0.268436578	Cross sectional	5
Guidoum et al. (75)	Algeria	2018.6–2019.8	ELISA	234	138	0.589743590	Cross sectional	5
Berg et al. (76)	Botswana	2014.10–2015.3	ELISA	364	195	0.535714286	Cross sectional	4
**Asia**								
Wang (77)	China	2011.6–2011.10	Neutralization	3503	1979	0.564944333	Cross sectional	3
Sha et al. (78)	China	2010–2012	ELISA	842	178	0.211401425	Cross sectional	3
Lin (79)	China	2014	ELISA	741	491	0.662618084	Cross sectional	4
Huang (80)	China	2015	ELISA	667	228	0.341829085	Cross sectional	3
Chen et al. (81)	China	2016.5–2016.12	ELISA	190	88	0.463157895	Cross sectional	3
Wang (82)	China	2016	ELISA	786	749	0.952926209	Cross sectional	3
Lu et al. (83)	China	2017.4–5	ELISA	150	74	0.493333333	Cross sectional	3
Sun et al. (84)	China	2016, 2017	ELISA	900	129	0.143333333	Cross sectional	3
Zhao et al. (85)	China	2018	ELISA	210	165	0.785714286	Cross sectional	3
Bi et al. (86)	China	2019.03–2020.02	ELISA	1601	969	0.605246721	Cross sectional	4
Fu et al. (87)	China	2010-2011	ELISA	1650	795	0.481818182	Cross sectional	3
Liu (88)	China	2011–2014	ELISA	522	333	0.637931034	Cross sectional	3
Liu (89)	China	2016.3–12	ELISA	559	202	0.361359571	Cross sectional	3
Cheng et al. (90)	China	2014–2016	ELISA	920	448	0.486956522	Cross sectional	3
Luo et al. (91)	China	2017.09–11	ELISA	897	179	0.199554069	Cross sectional	3
Zhu (92)	China	2017.03–12	ELISA	559	202	0.361359571	Cross sectional	3
Yan et al. (93)	China	2014–2015	UN	400	146	0.365000000	Cross sectional	2
Wang et al. (94)	China	2015.06–08	ELISA	191	124	0.649214660	Cross sectional	3
Zhao (95)	China	2014.09–2015.12	ELISA	326	294	0.902840491	Cross sectional	3
Cao et al. (96)	China	2014.04–2015.07	ELISA	86	17	0.197674419	Cross sectional	4
Liu et al. (97)	China	2011	ELISA	549	343	0.624772313	Cross sectional	4
Li et al. (98)	China	2012.06–12	ELISA	665	472	0.709774436	Cross sectional	4
Olmo et al. (99)	Laos	2016–2018	ELISA	520	30	0.057692308	Cross sectional	3
Noaman and Nabinejad (100)	Iran	2017.6–8	ELISA	216	114	0.527777778	Cross sectional	5
Li et al. (101)	China	2012–2014	ELISA	516	267	0.517441860	Cross sectional	5
Wang et al. (102)	China	2019–2020	ELISA	456	75	0.164473684	Cross sectional	4
Zhu (92)	China	2018.12–2019.3	ELISA	2358	1958	0.830364716	Cross sectional	3
Kumar et al. (103)	India	2014.9–2016.9	ELISA	500	66	0.132000000	Cross sectional	3
Zhong et al. (104)	China	2016	ELISA	604	113	0.187086093	Cross sectional	3
Chen et al. (105)	China	2014–2015	ELISA	1332	452	0.339339339	Cross sectional	3
Liu and Sun (106)	China	2012	ELISA	192	39	0.203125000	Cross sectional	3
Dong et al. (107)	China	2012	ELISA	492	244	0.495934959	Cross sectional	3
He et al. (108)	China	2012.9–2012.12	ELISA	1070	474	0.442990654	Cross sectional	3
Shang et al. (109)	China	2010–2012	ELISA	1198	282	0.235392321	Cross sectional	3
Han et al. (110)	China	2010.3–5	ELISA	252	54	0.214285714	Cross sectional	4
Ma et al. (111)	China	2013.4–2014.3	ELISA	1584	595	0.375631313	Cross sectional	3
Zhang et al. (112)	China	2012.01–2012.12	ELISA	460	237	0.515217391	Cross sectional	5
Liu et al. (113)	China	2016–2017	ELISA	597	296	0.495812395	Cross sectional	5
Luo (114)	China	2012–2016	ELISA	920	448	0.486956522	Cross sectional	4
Qu et al. (115)	China	2014.08–09	ELISA	1637	1013	0.618814905	Cross sectional	3
Xie et al. (116)	China	2014	ELISA	385	374	0.971428571	Cross sectional	5
Chen (117)	China	2016	ELISA	204	65	0.318627451	Cross sectional	4
Chen et al. (118)	China	2014.08–2015.02	ELISA	238	163	0.684873950	Cross sectional	5
Hu and Gu (119)	China	2015.09–2015.12	ELISA	917	450	0.490730643	Cross sectional	4
Cheng et al. (120)	China	2015.01–2015.06	ELISA	420	221	0.526190476	Cross sectional	4
Uddin et al. (121)	Bangladesh	2013.07–2014.04	ELISA	94	48	0.510638298	Cross sectional	5
Liu et al. (122)	China	2017.11–2018.4	ELISA	325	243	0.747700000	Cross sectional	4
Li et al. (123)	China	2019	ELISA	440	34	0.077272727	Cross sectional	5
Zhao (124)	China	2017.5–2018.10	ELISA	389	179	0.460154242	Cross sectional	4
Liu (125)	China	2018.12–2019.12	ELISA	1446	1244	0.860304288	Cross sectional	5
Liu et al. (125)	China	2017.11–2018.5	ELISA	792	518	0.654040404	Cross sectional	4
Shen et al. (126)	China	2010.5	ELISA	571	43	0.075306480	Cross sectional	3
Erfani et al. (127)	Iran	2011.12	ELISA	562	161	0.286000000	Cross sectional	5
Gan et al. (128)	China	2019.04–06	ELISA	455	36	0.079120879	Cross sectional	3
Kang et al. (129)	China	2012.5–2012.6	ELISA	546	14	0.025641026	Cross sectional	4
Lei et al. (130)	China	2011.11	ELISA	188	170	0.904255319	Cross sectional	4
Zhang et al. (131)	China	2012.11	ELISA	460	292	0.634782609	Cross sectional	4
Yuan et al. (132)	China	2012.6–2013.8	ELISA	244	144	0.590163934	Cross sectional	5
Liu (133)	China	2013	ELISA	566	247	0.436395760	Cross sectional	5
Yue et al. (134)	China	2013.7–2014.1	ELISA	266	102	0.383458647	Cross sectional	4
Yao (135)	China	2013–2014	ELISA	793	587	0.740226986	Cross sectional	5
Akagami et al. (136)	Japan	2014.4–2017.5	ELISA	9016	2378	0.263753327	Cross sectional	4
Singh et al. (137)	India	2013.10–2016.3	ELISA	466	71	0.152360515	Cross sectional	5
Katoch et al. (138)	India	2013–2015	ELISA	132	2	0.015151515	Cross sectional	5
Chowdhury et al. (139)	Bangladesh	2013.7–2014.4	UN	94	48	0.510638298	Cross sectional	3
Asnake et al. (140)	Bangladesh	2019.10–2020.4	ELISA	225	19	0.084444444	Cross sectional	5
Tadesse et al. (141)	Bangladesh	2016.1–2017.1	ELISA	420	217	0.516666667	Cross sectional	4
Daves et al. (142)	Malaysia	2014.11–2015.1	ELISA	407	135	0.331695332	Cross sectional	5
Manandhar et al. (143)	Nepal	2013.11–2014.4	ELISA	350	9	0.025714286	Cross sectional	4
Olmo et al. (144)	Laos	2013–2016	ELISA	151	12	0.079470199	Cross sectional	4
Nugroho et al. (21)	Indonesia	2017.3–7	ELISA	77	9	0.116883117	Cross sectional	3
**Europe**								
Rodríguez-Prieto et al. (145)	Spain	2008.9–2009.1	ELISA	180	82	0.455555556	Cross sectional	3
**North America**								
Segura-Correa et al. (146)	Mexico	2010.5–2011.12	ELISA	385	184	0.477922078	Cross sectional	5
**South America**								
Maya et al. (69)	Uruguay	2014	ELISA	390	298	0.764102564	Cross sectional	3

UN^*^: Unclear.

ELISA^*^: Enzyme linked immunosorbent assay (OIE).

Score^*^: Quality assessment (147).

**Table 4:** Antibody prevalence of Bovine viral diarrhea virus of cattle in the word.

	**No. studies**	**No. tested**	**No. positive**	% (95% CI^*^)	**Heterogeneity**			**Univariate meta-regression**	
					**χ^2^**	***P*-value**	**I^2^ (%)**	***P*-value**	**Coefficient (95% CI)**
**Area**									
Asia	71	53,457	23,597	42.03% (35.99–48.18)	14,291.55	0.00	99.5%	0.3912	−0.0960 (−0.3156– 0.1235)
Europe	1	180	82	45.56% (38.32–52.88)	0.00	–	–	
North America	1	385	184	47.79% (42.81–52.79)	0.00	–	–	
South America	1	390	298	76.41% (72.06–80.50)	0.00	–	–	
Africa	3	937	424	46.13% (26.82–66.06)	78.06	<0.01	97.4%	
**Sampling years**									
Before 2017	55	33,177	15,345	43.63% (37.25–50.13)	7,708.03	0.00	99.3%	0.7258	0.0251 (−0.1152– 0.1654)
After 2017	19	11,619	6,319	41.11% (27.05–55.95)	4,555.97	0.00	99.6%	
**Breed**									
Dairy	36	28,130	12,894	48.68% (39.19–58.22)	8,329.86	0.00	99.6%	0.0486	0.1329 (0.0008–0.2651)
Beef	11	3,176	1,020	29.46% (20.88–38.82)	266.77	<0.01	96.3%	
Both milk and meat	16	8,423	3,366	39.84% (30.54–49.53)	1,219.37	<0.01	98.8%	
**Health condition**									
Clinical Symptoms	11	3,889	2,266	58.66% (46.87–69.97)	452.44	<0.01	97.8%	0.1842	0.1460 (−0.0695– 0.3615)
Healthy	8	6,449	3,779	43.80% (26.57–61.83)	1,115.90	<0.01	99.4%	
**Season**									
Spring	6	2,335	1,103	46.70% (17.91–76.74)	1,165.27	<0.01	99.6%	
Summer	6	3,165	1,926	60.16% (48.92–70.89)	170.19	<0.01	97.1%	
Autumn	5	2,140	824	42.25% (18.14–68.51)	575.80	<0.01	99.3%	0.3256	−0.1417 (−0.4243– 0.1408)
Winter	5	1,836	995	63.44% (35.15–87.45)	605.47	<0.01	99.3%	
**Gender**									
Female	10	4,134	1,304	27.26% (19.11–36.24)	338.65	<0.01	97.3%	
Male	10	1,040	313	28.90% (17.71–41.45)	115.55	<0.01	92.2%	0.7922	0.0201 (−0.1296–0.1698)
**Age**									
Calf	18	4,883	2,307	38.25% (30.25–46.58)	422.11	<0.01	96.0%	
Adult cattle	31	27,636	12,889	48.82% (38.85–58.83)	7,625.49	0.00	99.6%	0.1677	0.1128 (−0.0475– 0.2731)
**Breeding mode**									
Intensive	55	39,104	18,857	50.35% (42.93–57.76)	11,248.83	0.00	99.5%	0.0067	0.2208 (0.0613–0.3803)
Extensive	11	3,530	1,093	23.58% (11.86–37.78)	762.24	<0.01	98.7%	
Mixed culture	4	553	261	44.75% (25.81–64.49)	60.59	<0.01	95.0%	
**Detection method** ^*^									
ELISA	74	51,352	22,412	42.56% (36.51–48.71)	14,306.05	0.00	99.5%	0.6068	−0.1396 (−0.6713– 0.3921)
Neutralization	1	3,503	1,979	56.49% (54.85–58.13)	0.00	–	–	
Total		77	55,349	24,585	42.77% (37.01–48.63)	14,553.30	0.00	99.5%	

CI^*^: Confidence interval.

Area^*^: Africa: Egypt, Ethiopia, Morocco; Asia: China, India; Europe: Italy, Spain, Portugal, France, Poland; North America: USA; Oceania: Australia; South America: Brazilian (147).

Method^*^: ELISA: Enzyme linked immunosorbent assay (OIE).

### 3.3. Meta-analysis based on detected antigen

**Figure 2:**
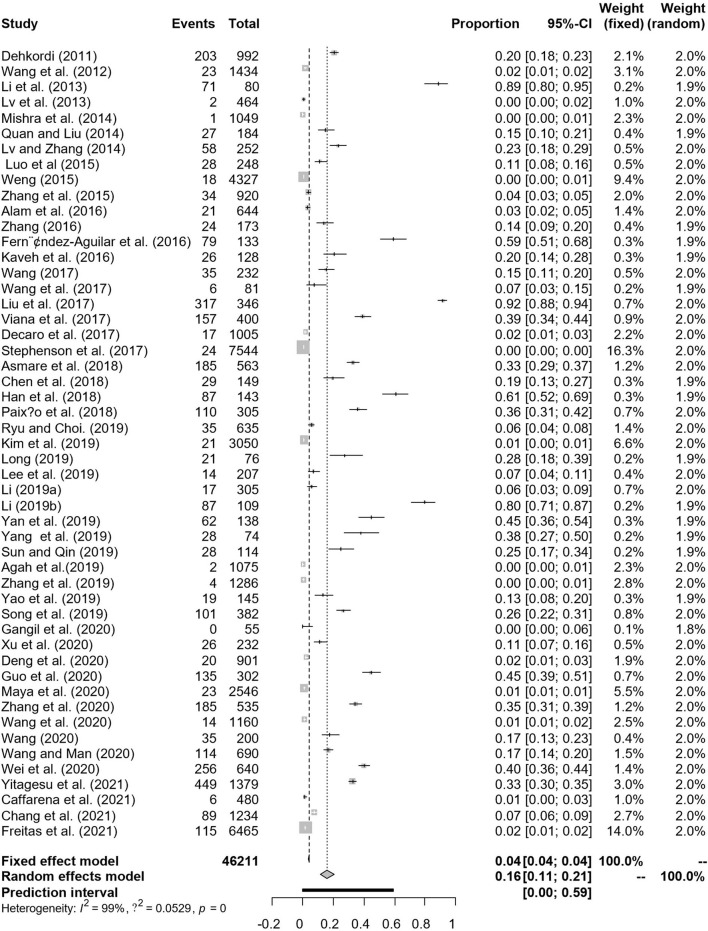
Forest plot of bovine viral diarrhea virus antigen prevalence in the world study conducted 2010–2021 (decetion antigen).

**Figure 3:**
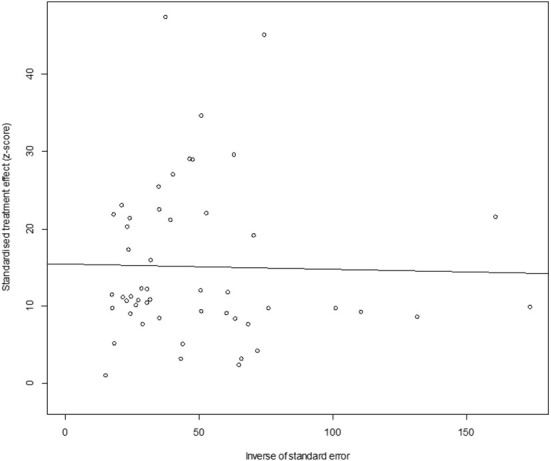
Egger's test for publication bias (decetion antigen).

**Figure 4:**
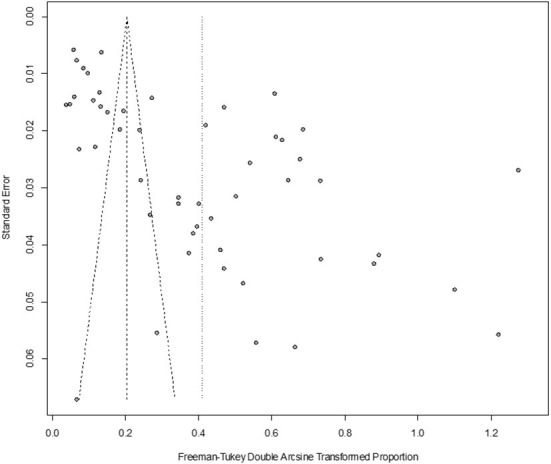
Funnel plot with pseudo 95% confidence interval limits for the examination of publication bias (decetion antigen).

In antigen detection, a total of 46,211 cattle were tested, and 3,488 BVDV-positive cattle were tested, with a positive infection rate of 15.74% (95% CI: 11.35–20.68, Figure 2). PFT conversion rate and random effect model (χ^2^ = 0.0566, I^2^ = 99%, *P* = 0.00) were used (Table 5). The egger test result is t = 6.5574, *p* = 6.975e−08 (Supplementary Table 3, Figure 3). The funnel diagram shows that there is bias (Figure 4). The trim and fill analysis are used to correct the bias, a total of 22 articles were corrected, and the adjusted prevalence rate was 2.01% (95% CI:0.40–4.64). The results of sensitivity analysis show that the results of meta-analysis are reliable (Table 6).

**Table 5:** Normal distribution test for the normal rate and the different conversion of the normal rate.

**Conversion form**	***W***	***P***
PRAW	0.81762	1.901e-06
PLN	NaN	NA
PLOGIT	NaN	NA
PAS	0.92342	0.002811
PFT	0.91785	0.001755

PRAW, original rate; PLN, logarithmic conversion; PLOGIT, logit transformation; PAS, arcsine transformation; PFT, double-arcsine transformation; NaN, meaningless number; NA, missing data.

**Table 6:** Sensitivity analysis (decetion antigen).

**Reference ID**	**% (95% CI)**
Omitting Dehkordi (58)	15.65% (11.25–20.61)
Omitting Wang et al. (31)	16.16% (11.62–21.29)
Omitting Li et al. (98)	14.61% (10.41–19.38)
Omitting Lv et al. (148)	16.24% (11.73–21.31)
Omitting Mishran et al. (61)	16.30% (11.77–21.39)
Omitting Quan and Liu (45)	15.76% (11.33–20.76)
Omitting Lv and Zhang (46)	15.60% (1120–20.57)
Omitting Luo et al. (44)	15.83% (11.39–20.85)
Omitting Weng (59)	16.26% (11.64–21.48)
Omitting Zhang et al. (60)	16.06% (11.55–21.15)
Omitting Alam et al. (63)	16.08% (11.57–21.15)
Omitting Zhang (43)	15.78% (11.34–20.78)
Omitting Fernández–Aguilar et al. (66)	15.07% (10.78–19.92)
Omitting Kaveh et al. (48)	15.65% (11.24–20.63)
Omitting Wang (34)	15.75% (11.32–20.75)
Omitting Wang et al. (35)	15.92% (11.47–20.94)
Omitting Liu et al. (47)	14.45% (10.54–18.85)
Omitting Viana et al. (71)	15.34% (11.02–20.21)
Omitting Decaro et al. (67)	16.16% (11.63–21.25)
Omitting Stephenson et al. (73)	16.28% (11.62–21.55)
Omitting Asmare et al. (65)	15.44% (11.10–20.32)
Omitting Chen et al. (36)	15.67% (11.25–20.65)
Omitting Han et al. (110)	15.04% (10.76–19.89)
Omitting Paixão et al. (70)	15.39% (11.05–20.30)
Omitting Ryu and Choi (41)	15.99% (11.50–21.06)
Omitting Kim et al. (42)	16.23% (11.63–21.42)
Omitting Long (30)	15.54% (11.15–20.50)
Omitting Lee et al. (32)	15.95% (11.49–20.98)
Omitting Li (33)	15.99% (11.51–21.03)
Omitting Li (37)	14.76% (10.53–19.54)
Omitting Yan et al. (38)	15.27% (10.93–20.17)
Omitting Yang et al. (38)	15.39% (11.02–20.32)
Omitting Sun and Qin (39)	15.58% (11.18–20.55)
Omitting Agah et al. (52)	16.28% (11.76–21.37)
Omitting Zhang et al. (53)	16.27% (11.73–21.37)
Omitting Yao et al. (54)	15.79% (11.36–20.79)
Omitting Song et al. (55)	15.54% (11.16–20.49)
Omitting Gangil et al. (62)	16.22% (11.74–21.27)
Omitting Xu et al. (25)	15.84% (11.39–20.85)
Omitting Deng et al. (26)	16.13% (11.61–21.22)
Omitting Guo et al. (27)	15.26% (10.96–20.12)
Omitting Maya et al. (69)	16.21% (11.63–21.39)
Omitting Zhang et al. (29)	15.41% (11.08–20.29)
Omitting Wang et al. (49)	16.19% (11.65–21.29)
Omitting Wang (50)	15.70% (11.28–20.69)
Omitting Wang and Man (56)	15.72% (11.29–20.72)
Omitting Wei et al. (57)	15.32% (11.05–20.14)
Omitting Yitagesu et al. (64)	15.43% (11.19–20.20)
Omitting Caffarena et al. (68)	16.18% (11.67–21.24)
Omitting Chang et al. (89)	15.95% (11.44–21.04)
Omitting Freitas et al. (72)	16.17% (11.41–21.57)

### 3.4. Meta-analysis based on detected antibody

**Figure 5:**
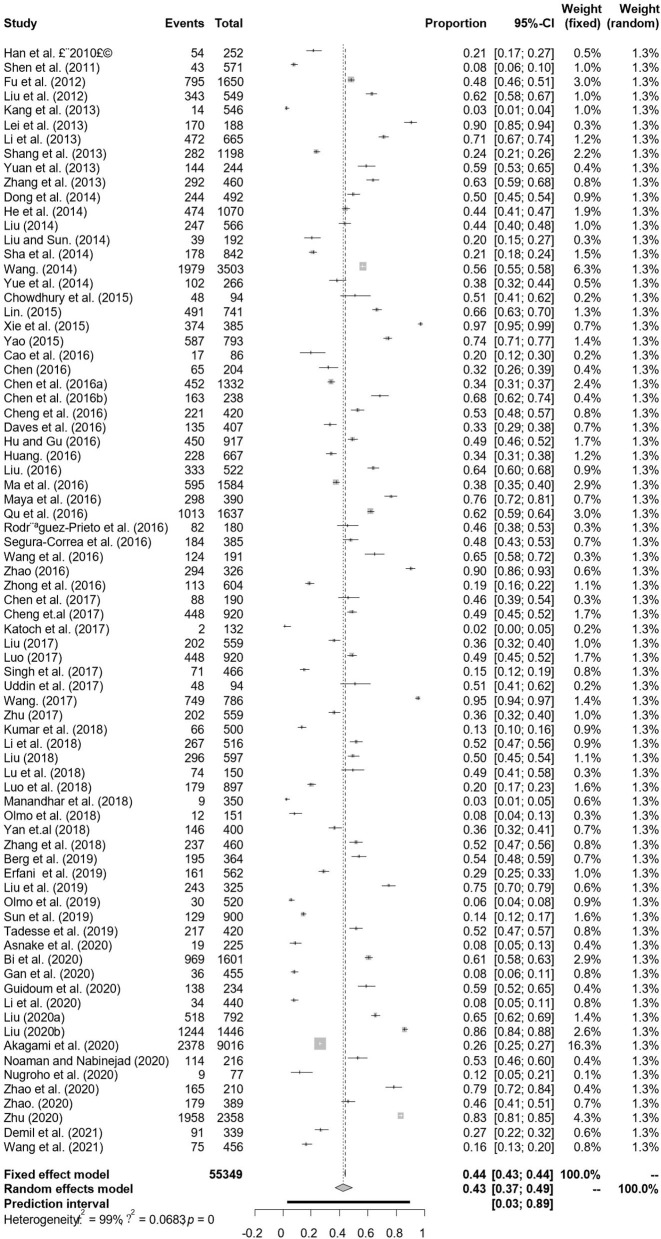
Forest plot of bovine viral diarrhea virus antibody prevalence in the world study conducted 2010–2021 (decetion antibody).

**Figure 6:**
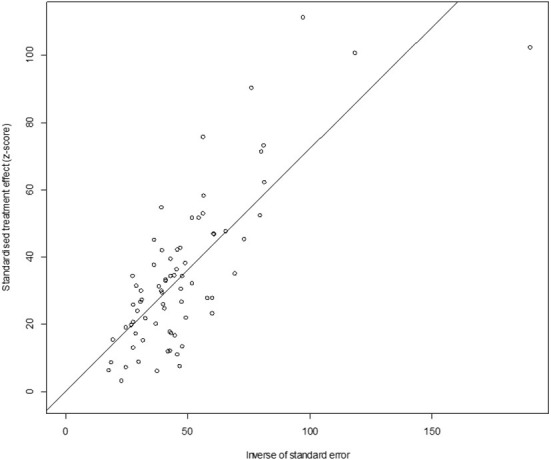
Egger's test for publication bias (decetion antibody).

**Figure 7:**
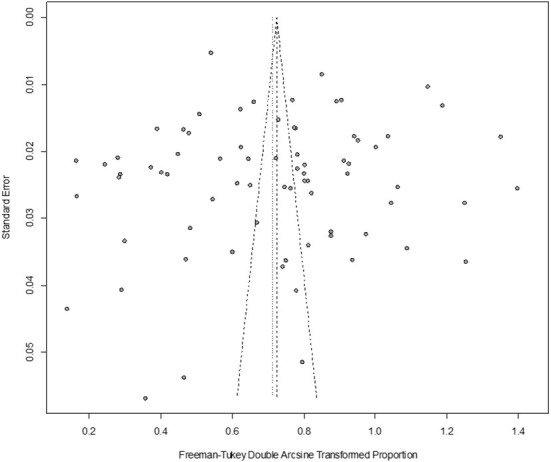
Funnel plot with pseudo 95% confidence interval limits for the examination of publication bias (decetion antibody).

In antibody detection, a total of 55,349 samples were tested, of which 24,585 were positive, and the positive rate was 42.77% (95% CI: 37.01–48.63, Figure 5). PFT conversion rate and random effect model(χ^2^ = 0.0664, I2 = 100%, *P* = 0.00, Table 7) were used. The egger test result is t = 0.68873, *p* = 0.4935 (Supplementary Table 4, Figure 6). The funnel diagram shows that there is bias (Figure 7). The data from the trim and fill analysis showed that no trimming performed, and the data unchanged, meaning there may be no significant publication bias. The results of sensitivity analysis show that the results of meta-analysis are reliable (Table 8).

**Table 7:** Normal distribution test for the normal rate and the different conversion of the normal rate.

**Conversion form**	***W***	***P***
PRAW	0.97216	0.0889
PLN	0.85225	2.918e-07
PLOGIT	0.97702	0.1767
PAS	0.98413	0.4527
PFT	0.98468	0.4835

PRAW, original rate; PLN, logarithmic conversion; PLOGIT, logit transformation; PAS, arcsine transformation; PFT, double-arcsine transformation; NaN, meaningless number; NA, missing data.

**Table 8:** Sensitivity analysis (decetion antibody).

**Reference ID**	**% (95% CI)**
Omitting Han et al. (110)	43.07% (37.26–48.97)
Omitting Shen et al. (126)	43.34% (37.58–49.18)
Omitting Fu et al. (87)	42.69% (36.81–48.69)
Omitting Liu et al. (97)	42.51% (36.70–48.42)
Omitting Kang et al. (129)	43.49% (37.78–49.28)
Omitting Lei et al. (130)	42.07% (36.32–47.93)
Omitting Li et al. (51)	42.39% (36.60–48.28)
Omitting Shang et al. (109)	43.04% (37.21–48.96)
Omitting Yuan et al. (132)	42.56% (36.75–48.46)
Omitting Zhang et al. (131)	42.49% (36.69–48.40)
Omitting Dong et al. (107)	42.68% (36.85–48.60)
Omitting He et al. (108)	42.75% (36.89–48.71)
Omitting Liu (133)	42.76% (36.93–48.69)
Omitting Liu and Sun (106)	43.08% (37.28–48.99)
Omitting Sha et al. (78)	43.08% (37.26–48.98)
Omitting Wang. (77)	42.58% (36.65–48.63)
Omitting Yue et al. (134)	42.83% (37.01–48.74)
Omitting Chowdhury et al. (139)	42.66% (36.86–48.56)
Omitting Lin (79)	42.45% (36.65–48.36)
Omitting Xie et al. (116)	41.88% (36.22–47.64)
Omitting Yao (135)	42.34% (36.57–48.22)
Omitting Cao et al. (96)	43.08% (37.28–48.98)
Omitting Chen (117)	42.91% (37.10–48.82)
Omitting Chen et al. (105)	42.89% (37.02–48.85)
Omitting Chen et al. (118)	42.43% (36.64–48.33)
Omitting Cheng et al. (120)	42.64% (36.82–48.56)
Omitting Daves et al. (142)	42.90% (37.08–48.82)
Omitting Hu and Gu (119)	42.68% (36.84–48.63)
Omitting Huang (149)	42.88% (37.05–48.82)
Omitting Liu (88)	42.49% (36.69–48.40)
Omitting Ma et al. (111)	42.84% (36.95–48.82)
Omitting Maya et al. (69)	42.31% (36.54–48.19)
Omitting Qu et al. (115)	42.51% (36.67–48.46)
Omitting Rodríguez-Prieto et al. (145)	42.73% (36.92–48.64)
Omitting Segura-Correa et al. (146)	42.70% (36.88–48.62)
Omitting Wang et al. (94)	42.48% (36.68–48.38)
Omitting Zhao (95)	42.07% (36.34–47.90)
Omitting Zhong et al. (104)	43.11% (37.31–49.01)
Omitting Chen et al. (81)	42.72% (36.91–48.63)
Omitting Cheng et al. (90)	42.69% (36.84–48.64)
Omitting Katoch et al. (138)	43.51% (37.72–49.38)
Omitting Liu (89)	42.86% (37.03–48.79)
Omitting Luo (114)	42.69% (36.84–48.64)
Omitting Singh et al. (137)	43.17% (37.38–49.06)
Omitting Uddin et al. (121)	42.66% (36.86–48.56)
Omitting Wang (34)	41.93% (36.36–47.61)
Omitting Zhu (105)	42.86% (37.03–48.79)
Omitting Kumar et al. (103)	43.21% (37.43–49.09)
Omitting Li (101)	42.65% (36.83–48.57)
Omitting Liu (113)	42.68% (36.85–48.61)
Omitting Lu et al. (83)	42.68% (36.88–48.59)
Omitting Luo et al. (91)	43.10% (37.29–49.00)
Omitting Manandhar et al. (143)	43.48% (37.74–49.32)
Omitting Olmo et al. (144)	43.31% (37.52–49.20)
Omitting Yan et al. (93)	42.85% (37.03–48.77)
Omitting Zhang et al. (112)	42.65% (36.83–48.57)
Omitting Berg et al. (76)	42.63% (36.81–48.54)
Omitting Erfani et al. (127)	42.96% (37.14–48.88)
Omitting Liu et al. (122)	42.34% (36.56–48.22)
Omitting Olmo et al. (99)	43.38% (37.64–49.22)
Omitting Sun et al. (84)	43.19% (37.42–49.06)
Omitting Tadesse et al. (141)	42.65% (36.83–48.57)
Omitting Asnake et al. (140)	43.31% (37.52–49.19)
Omitting Bi et al. (86)	42.53% (36.69–48.48)
Omitting Gan et al. (128)	43.33% (37.56–49.18)
Omitting Guidoum et al. (75)	42.56% (36.75–48.46)
Omitting Li et al. (123)	43.33% (37.56–49.19)
Omitting Li et al. (123)	42.47% (36.66–48.38)
Omitting Liu (125)	42.14% (36.54–47.85)
Omitting Akagami et al. (136)	42.99% (37.05–49.04)
Omitting Noaman and Nabinejad (100)	42.64% (36.83–48.55)
Omitting Nugroho et al. (21)	43.22% (37.41–49.11)
Omitting Zhao et al. (85)	42.28% (36.51–48.16)
Omitting Zhao (124)	42.72% (36.91–48.65)
Omitting Zhu (105)	42.20% (36.65–47.85)
Omitting Demil et al. (74)	42.99% (37.17–48.90)
Omitting Wang et al. (102)	43.15% (37.35–49.05)

## 4. Discussion

BVDV is one of the most important bovine infectious diseases with global animal health and economic impacts. BVDV infection will not only cause huge economic losses to the breeding industry, but also in animal research and medical industry related serum, vaccines and other biological not infected with BVDV but contaminated with BVDV, which has a huge economic impact (150). BVDV can be spread in many ways. BVDV is widely transmitted, not only through direct contact, but also through various excreta, contaminated materials, etc (151). However, vertical transmission plays an important role in its epidemiology and pathogenesis. PI calves produced by pregnant cows through vertical transmission are the main source of infection of the disease, and they continue to be infected and carry BVDV pathogens throughout their lives. PI cattle are the main host of the virus. A large number of viruses are excreted from urine, feces, excrement, milk and semen, causing serious obstacles to the control of the disease.

The article searched all articles on the epidemiology of bovine BVDV in 2010–2021. The meta-analysis included 128 articles. Through the analysis, it is expected to investigate the latest data on the global prevalence of BVDV and provide data support for the prevention and control of BVDV. The detection of BVDV is usually divided into the detection of antigens and the detection of antibodies. A positive antigen represents the current prevalence of animals carrying BVDV pathogens, making it clear that the virus is spreading and harming the population. Positive antibody indicates infection, vaccine immunization or transient infection. As individuals immunized with vaccine are excluded, positive antibody in the article can be considered as being infected by virus. Both have important guiding significance for the description of the BVDV epidemic.

In the regional subgroup, there were fewer test samples in Europe, possibly due to large-scale vaccinations in Europe and not included in the study; On the other hand, it may be due to the fact that many European countries have eradicated BVDV or the prevalence rate has dropped to 1.5% (152). Examples include Denmark, Norway, Sweden and Finland (153). Switzerland, Austria and Germany are in the late or final stages of eradicating BVDV, followed by plans to eradicate BVDV in the Netherlands, Ireland and Poland (19). Control measures in several countries are mainly aimed at the clearance of PI animals. As early as 1990, a non-vaccination program in the Scandinavian countries was implemented to eliminate BVDV, which was planned to detect and remove PI animals based on ear groove samples (154). The Swiss clearance program restricts action mainly on pregnant cattle and directly tests for antigenic and viral genomes (155). Ireland's clearance program focuses on monitoring ear groove samples from newborn calves (156). PI animals are immune to BVDV and are unable to develop specific antibodies against it, which increases the obstacle to virus clearance and is also the main source of infection of the disease (157). And the mutagenicity of the virus itself, as well as the infection of cp BVDV from the outside world, has developed into a fatal mucosal disease, causing serious harm to the herd (158). There is evidence that when PI animals disappear, population virus transmission is largely stopped. However, the impact of removing only PI without considering TI is still debatable. There are cases where BVDV will persist for 6 years without PI mavericks (159). The successful implementation of a BVDV control plan should consider the impact of both modes of infection. In the process of removing PI, the prevalence rate should be monitored at the same time, and TI animals should be monitored in a timely manner. While the prevalence of PI animals varies from region to region in terms of legal support, it took nearly 10 years for all countries to reach the final stages of the control plan (160, 161). The long-term implementation of the plan also suggests that in order to successfully complete the purification, strict policies, strict management, and a high degree of prevention awareness of practitioners are required.

In the regional subgroups, the low prevalence rate in North America and the high prevalence rate in South America and South America may result in a small number of articles and be unrepresentative due to the limitations of search. There is no data on antigen testing in Africa, while the prevalence of antibody testing remains high. This also reminds us that although many African countries have carried out surveillance and culling of BVDV, it will take a long time to eliminate BVDV. Asia has the largest literature and the infection rate remains high, with no significant differences in regional subgroups. The high infection rate in Asia may be due to the lack of a sound control plan and a surge in herd numbers due to the rapid development of the cattle industry. From the measures in different regions, we can find that the control plan in Poland suggests that it is very important to control the possible risk of virus transmission if the eradication plan is to be successful. From the German plan, it can be found that voluntary policies are not enough to achieve freedom from disease, and the initial implementation of voluntary policies eventually leads to mandatory plans (19). The control plan of the Netherlands points out that in areas where BVDV has been eradicated, the increase of susceptible animals makes the area more affected by BVDV, so timely detection should be carried out to reduce the possibility of transmission of BVDV (162). Monitoring plays an important role in reducing the spread of BVDV, and the comprehensiveness of the sample survey is critical to the success of the eradication plan (20). It can be concluded from the control and eradication plans implemented in different countries and regions that the identification and isolation of PI animals is the key to the eradication plan, and vaccination and appropriate safety measures are the basic methods of the control plan (163). Therefore, countries and areas that have implemented eradication plans should conduct timely and regular prevalence surveys. Other areas should implement corresponding eradication plans as soon as possible.

Due to different control measures in different regions, a subgroup analysis of time for global BVDV antigen testing and antibody testing found that the prevalence after 2017 did not decrease significantly compared with the prevalence before 2017. Previous articles have analyzed that in the global region, the prevalence of PI showed a downward trend from 1982 to 2016, while the level of antibody prevalence was relatively stable (164). Our data also shows that the prevalence of antigens and antibodies has remained relatively stable since 2017. It is suggested that we should improve the corresponding eradication policy and give certain time and patience to eliminate pathogens. BVDV still has a high infection rate, and this high spread may be due to the lack of complete prevention and control measures for BVDV, the most important reason being the failure to detect and eliminate PI animals (151). In addition, the lack of commercial vaccines and reasonable and effective prevention and control programs is one of the reasons for the high prevalence of BVDV (27). Commercial transportation, fertilization of breeding cattle, and the introduction of new herds are all indispensable factors in the spread of disease, hindering the eradication of BVDV. Therefore, it is important to improve the monitoring of BVDV and introduce relevant control measures (151, 165).

In the breed subgroup, the antibody test results were the highest among dairy cattle, with significant difference. On the one hand, it may be due to the long service period of dairy cows, which have more opportunities to contact with pathogens. Studies have also confirmed that the positive rates of tuberculosis, brucellosis and bovine leukemia virus in dairy cows are higher than those in beef cattle (165, 166). On the other hand, compared with beef cattle, there may be more contact between cows and milkers, and the cross-infection among different cows is more intensive during milking, which leads to more opportunities for virus contact and greater risk of infection (142). Among the antigen test results, the positive rate of dairy cows was the lowest, and the difference was significant. Perhaps this is because the harm of antigen-positive cows to dairy cows is more intuitive, such as decreased milk yield, stillbirth, abortion, etc (12, 167, 168), and the production performance of dairy cows needs higher health, so antigen-positive cows are eliminated in time (169–171). For different breeds of cattle, different control measures should be taken according to different economic uses and lifestyles, and strict management should be taken to reduce the prevalence of BVDV.

Age has long been considered the most common influencing factor associated with infection rates (172). The data in the article show that the prevalence of adult cattle is higher than that of calve, and there is no significant difference. Many studies have also pointed out that the prevalence of adult cattle is higher than that of calves as a factor of age (74, 149, 173). This may be due to the longer survival time of adult cattle, a higher chance of exposure to the virus, and a higher probability of infection (141). In addition, antibody prevalence in calves is much higher than antigen prevalence, possibly due to the fact that calves can obtain colostrum antibodies from the mother (174). For calves, whether they are PI cattle carrying antigens should be detected in time, and eliminated in time to prevent the spread of infection. Adult cattle should have a reasonable detection system and a sound management system to reduce the chance of contact with the virus and reduce the prevalence of the disease.

The survey data of the article shows that the prevalence rate is relatively high in winter and spring, no matter for antigen detection or antibody detection. This may be due to the breeding season in spring and winter. It has been reported that in winter and spring, both male and female animals have strong reproductive performance, which is more conducive to cattle breeding (175, 176). The spread of BVDV in the breeding process led to the birth of more PI positive calves, further promoting the increase of the positive rate. The prevalence of BVDV antibody is still high in summer. It may be that the PI animals produced continuously expel viruses to the external environment, leading to the expansion of infection. Some literature points out that there is no antiviral drug to prevent the spread of BVDV in the farm at present, and the spread of the virus can only be prevented by isolating PI animals or vaccinating (177). Therefore, BVDV detection should be done well in the breeding season to reduce the production of PI animals and control the spread of BVDV from the source.

Many articles point out that gender does not have a large influence on infection rates, and bulls are just as susceptible as cows (74). The results of this survey show that there is no significant difference, which is consistent with other research results. Investigation samples of cows are much larger than those of bulls, possibly due to the fact that bulls are mostly used as beef cattle and female cows are used for milk production and reproductive purposes. Female cattle are more affected by the disease. Since PI calves born of cow infection during the first trimester of pregnancy are the main source of infection of the disease, cow test samples are collected in antigen testing to prevent the birth of PI cattle. Therefore, the prevention and control of PI cattle can be screened for antigens from pregnant cows.

In the introduction of BVDV 2018, the diagnosis of BVDV includes nucleic acid detection of QPCR, antigen antibody detection of ELISA, IHC, VN and virus isolation. PCR method can almost meet all purposes of detection, including making group animals free from infection, individual animals free from infection before moving, promoting the implementation of eradication policy, confirmation of clinical cases and detection of infection rate. PCR detection method has the advantages of convenience, rapidity and large sample size. The ELISA method is not applicable to animals with acute infection. IHC is mainly for diagnostic investigation. VN and virus isolation are usually used in laboratory research. It can be seen in the antigen detection data that the positive rate of PCR test is higher than the positive rate of ELISA test. There have also been reports of low sensitivity and accuracy of ELISA testing compared to PCR (178). Young animals can also obtain BVDV antibodies from the milk of female animals, thereby reducing the detection rate of ELISA antigens. This is consistent with the findings of this paper. VN has the highest detection rate, but it is difficult to detect, the sample detection volume is small, and it is generally not used in epidemiological investigations (28). In epidemiological investigations, antibody testing mostly chooses the rapid and inexpensive ELISA test, while the antigen test chooses a more sensitive PCR test (179).

The results of the survey show that most of the antibody test samples are derived from serum, and the test is relatively mature. The feces positivity rate was highest among the antigen-tested samples, followed by blood samples and ear tissue sample. The ear tissue sample is significantly different from other samples, and its low prevalence rate may be due to the fact that the sample was collected from the area where BVDV purification was carried out. Feces samples and blood samples can reflect the prevalence of infection. Feces sample collection is more convenient, the harm to cattle is small, but there is a possibility of cross-infect; Stress may occur on cattle during blood sample collection; Ear tissue samples are often used for the removal of PI cattle under the purification policy of various regions. For the detection of BVDV in cattle, the most appropriate samples shall be taken according to local conditions to make the detection results comprehensive and correct (180).

Breeding mode has always been a key factor affecting BVDV infection. An article survey shows that the low prevalence of grazing and breeding is due to the low density of grazing and breeding (40). However, some data show that the prevalence of intensive farming is low, and some studies show that grazing and breeding have the opportunity to contact more pathogens. Studies have pointed out that although BVDV cannot be transmitted by flies, flies have been shown to carry the BVDV pathogen (181). In the breeding mode subgroup, the positive rate of intensive culture was higher than that of extensive culture. On the one hand, there may be errors in monitoring the infection rate due to the difficulty in sampling free range animals. On the other hand, the virus may spread widely due to the high density of intensive farming. In addition, BVDV is introduced and spread through contaminated houses, water tanks, feeds and feeding equipment (182).

When BVDV infection occurs, the clinical symptoms of acute infected animals usually include temperature rise to 40°C, diarrhea, oral erosion, etc. There are few or no clinical symptoms observed in other infections (183). Mucosal diseases induced by BVDV do not show clinical symptoms within 1 week. After 1 week, severe diarrhea, dehydration, anorexia, and lethargy will occur, and death will occur 1 week after clinical symptoms (184). Due to the low incidence of acute infection and mucosal disease, BVDV infection will not lead to obvious clinical infection, or only non-specific clinical symptoms and immunosuppression (6). The immunosuppression will be secondary to other pathogenic infections, which may cause a series of clinical symptoms and endanger the health level of livestock (185). Through the division of clinical health, the results showed that the prevalence rate of cattle with clinical symptoms was higher than that of clinical healthy cattle, indicating that regular detection of cattle health was also an important way to prevent and control infection. Therefore, whether or not having a sound management system is the key to affecting the infection rate of the intensive breeding industry. A reasonable and perfect management system can greatly reduce the spread of virus.

Through our meta-analysis, we found that the prevalence of BVDV in the world is still very high. In the areas where the eradication plan is implemented, attention should still be paid to controlling the possible transmission risk of the virus. In terms of time span, the control and elimination of BVDV requires the joint efforts of all countries and regions to develop reasonable and effective prevention and control programs to eliminate PI animals. At the same time, the elimination of BVDV requires a certain degree of patience, timely grasp the epidemic situation, and improve the prevention and control policy. Different control measures should be taken for different breeds of cattle, and strict management policies are required to reduce BVDV infection. After calves are born, they should be tested for antigen in time to reduce the birth of PI cattle. BVDV detection and elimination should be done well in winter and spring breeding seasons. For cows, it is necessary to timely detect whether there is antigen infection before pregnancy to prevent the production of PI cattle. The ear tissue samples selected for antigen monitoring are more accurate, VN detection method has a higher accuracy, while PCR detection method has a wide detection range and a large sample detection volume. Generally, ELISA is used to detect serum samples. In raising cattle, attention should be paid to the cleanliness and hygiene of the breeding environment.

To sum up, based on the epidemiological situation of BVDV in different areas, the eradication and prevention policies should be formulated and revised in time. Meanwhile, it is necessary to strengthen the awareness of herders to diseases and increase the awareness of veterinary and other related professionals to prevent and control BVDV. Our meta-analysis still has some limitations. The main reasons are as follows: 1. Due to the choice of language and database, it was not included in all studies. 2. The data cannot be downloaded or excluded from the inclusion and exclusion criteria. 3. Many countries do not have perfect testing procedures and do not test all cattle.

## Data availability statement

The original contributions presented in the study are included in the article/Supplementary material, further inquiries can be directed to the corresponding authors.

## Author contributions

RD and KS: idea and concept. NS: writing and editing of the manuscript. RD: funding. H-YL, L-ML, QW, and KS: revision of the manuscript. Q-XM, T-TW, WZ, and T-LY: collection and extraction of data. TT and J-YY: database establishment. TL, N-CD, QW, and J-ML: data analysis. All the authors contributed to the editing of the manuscript and approved the final manuscript.
