# Supplementary material for: Prevalence of bovine viral diarrhea virus in cattle between 2010 and 2021: A global systematic review and meta-analysis
Source: Front Vet Sci. 2023 Jan 17;9:1086180. doi: 10.3389/fvets.2022.1086180 (PMC9887317; doi:10.3389/fvets.2022.1086180)
Supplement: Supplementary file 1 [file Table_1.docx]

**Supplementary Material 1.** PRISMA Checklist item.

| **Section/topic** | **#** | **Checklist item** | **Reported on page #** |
| --- | --- | --- | --- |
| **TITLE** |  |  |  |
| Title | 1 | Prevalence of Bovine Viral Diarrhea Virus in Cattle between 2010 and 2021: A Global Systematic Review and Meta-Analysis | 1 |
| **ABSTRACT** |  |  |  |
| Structured summary | 2 | Background: Bovine viral diarrhea is one of the diseases that cause huge economic losses in animal husbandry. Many countries or regions have successively introduced eradication plans, but BVDV still has a high prevalence in the world. This meta-analysis aims to investigate the prevalence and risk factors of BVDV in the world in recent 10 years, and is expected to provide some reference and theoretical basis for BVDV control plans in different regions.  Method: Relevant articles published from 2010 to 2021 were mainly retrieved from NCBI, ScienceDirect, Chongqing VIP, Chinese web of knowledge (CNKI), web of science and Wanfang databases.  Results: 128 data were used to analyze the prevalence of BVDV from 2010 to 2021. BVDV antigen prevalence rate is 15.74% (95% CI: 11.35-20.68), antibody prevalence rate is 42.77% (95% CI: 37.01-48.63). In the two databases of antigen and antibody, regions, sampling time, samples, detection methods, species, health status, age, sex, breeding mode, and seasonal subgroups were discussed and analyzed respectively. In the antigen database, the prevalence of dairy cows in the breed subgroup, ELISA in the detection method subgroup, ear tissue in the sample subgroup, and extensive breeding in the breeding mode were the lowest, with significant differences. In the antibody database, the prevalence rate of dairy cows in the breed subgroup and intensive farming was the highest, with a significant difference. The subgroups in the remaining two databases were not significantly different.  Conclusion: This meta-analysis determined the prevalence of BVDV in global cattle herds from 2010 to 2021. The prevalence of BVDV varies from region to region, and the situation is still not optimistic. In daily feeding, we should pay attention to the rigorous and comprehensive management to minimize the spread of virus. The government should enforce BVDV prevention and control, implement control or eradication policies according to local conditions, and adjust the policies in time. | 2-3 |
| **INTRODUCTION** |  |  |  |
| Rationale | 3 | Describe the rationale for the review in the context of what is already known. | 3 |
| Objectives | 4 | Provide an explicit statement of questions being addressed with reference to participants, interventions, comparisons, outcomes, and study design (PICOS). | 3-5 |
| **METHODS** |  |  |  |
| Protocol and registration | 5 | Indicate if a review protocol exists, if and where it can be accessed (e.g., Web address), and, if available, provide registration information including registration number. | 5-7 |
| Eligibility criteria | 6 | Specify study characteristics (e.g., PICOS, length of follow-up) and report characteristics (e.g., years considered, language, publication status) used as criteria for eligibility, giving rationale. | 5-7 |
| Information sources | 7 | Describe all information sources (e.g., databases with dates of coverage, contact with study authors to identify additional studies) in the search and date last searched. | 5-7 |
| Search | 8 | Present full electronic search strategy for at least one database, including any limits used, such that it could be repeated. | 5-7 |
| Study selection | 9 | State the process for selecting studies (i.e., screening, eligibility, included in systematic review, and, if applicable, included in the meta-analysis). | 4 |
| Data collection process | 10 | Describe method of data extraction from reports (e.g., piloted forms, independently, in duplicate) and any processes for obtaining and confirming data from investigators. | 3-5 |
| Data items | 11 | List and define all variables for which data were sought (e.g., PICOS, funding sources) and any assumptions and simplifications made. | 4-6 |
| Risk of bias in individual studies | 12 | Describe methods used for assessing risk of bias of individual studies (including specification of whether this was done at the study or outcome level), and how this information is to be used in any data synthesis. | 4-6 |
| Summary measures | 13 | State the principal summary measures (e.g., risk ratio, difference in means). | 4 |
| Synthesis of results | 14 | Describe the methods of handling data and combining results of studies, if done, including measures of consistency (e.g., I^2^) for each meta-analysis. | 4-6 |
| Risk of bias across studies | 15 | Specify any assessment of risk of bias that may affect the cumulative evidence (e.g., publication bias, selective reporting within studies). | 4-6 |
| Additional analyses | 16 | Describe methods of additional analyses (e.g., sensitivity or subgroup analyses, meta-regression), if done, indicating which were pre-specified. | 4-6 |
| **RESULTS** |  |  |  |
| Study selection | 17 | Give numbers of studies screened, assessed for eligibility, and included in the review, with reasons for exclusions at each stage, ideally with a flow diagram. | 4-6, Table 1, Figure 1 and 2 |
| Study characteristics | 18 | For each study, present characteristics for which data were extracted (e.g., study size, PICOS, follow-up period) and provide the citations. | 4-6, Table 2, Figure 2 |
| Risk of bias within studies | 19 | Present data on risk of bias of each study and, if available, any outcome level assessment (see item 12). | 5-6, Figure 3, Figure 4, Figure 5, Figure 6and Figure S3-9 |
| Results of individual studies | 20 | For all outcomes considered (benefits or harms), present, for each study: (a) simple summary data for each intervention group (b) effect estimates and confidence intervals, ideally with a forest plot. | 5-6, Table 3, Figure 7 |
| Synthesis of results | 21 | Present results of each meta-analysis done, including confidence intervals and measures of consistency. | 5-6, Figure 2 |
| Risk of bias across studies | 22 | Present results of any assessment of risk of bias across studies (see Item 15). | 5-6, Table 3 |
| Additional analysis | 23 | Give results of additional analyses, if done (e.g., sensitivity or subgroup analyses, meta-regression [see Item 16]). | 5-6, Table 3 |
| **DISCUSSION** |  |  |  |
| Summary of evidence | 24 | Summarize the main findings including the strength of evidence for each main outcome; consider their relevance to key groups (e.g., healthcare providers, users, and policy makers). | 6-9 |
| Limitations | 25 | Discuss limitations at study and outcome level (e.g., risk of bias), and at review-level (e.g., incomplete retrieval of identified research, reporting bias). | 6-9 |
| Conclusions | 26 | Provide a general interpretation of the results in the context of other evidence, and implications for future research. | 6-9 |
| **FUNDING** |  |  |  |
| Funding | 27 | Describe sources of funding for the systematic review and other support (e.g., supply of data); role of founders for the systematic review. | 10 |

*From:* Moher D, Liberati A, Tetzlaff J, Altman DG, The PRISMA Group (2009). Preferred Reporting Items for Systematic Reviews and Meta-Analyses: The PRISMA Statement. PLoS Med 6(6): e1000097. doi:10.1371/journal.pmed1000097

For more information, visit: **www.prisma-statement.org**.

**Supplementary Material 2.** The code in R for meta-analysis.

| Logarithmic conversion (PNL) | rate<-transform [m1, log=log(event/n)];  shapiro.test(rate$log) |
| --- | --- |
| Logit transformation (PLOGIT) | rate<-transform{m1, logit=log[(event/n)/(1-event/n)]};  shapiro.test(rate$logit) |
| Arcsine transformation (PAS) | rate<-transform{m1, arcsin.size=asin[sqrt(event/(n+1))]};  shapiro.test(rate$arcsin) |
| Double-arcsine transformation (PFT) | rate<-transform{m1,darcsin=0.5*[asin(sqrt(event/(n+1)))+asin((sqrt(event+1)/(n+1)))]};  shapiro.test(rate$darcsin) |
| No transformation (PRAW) | rate<-transform[m1, r= event/n];  shapiro.test(rate$r) |
| Forest plots | forest [meta1, xlim=c(-0.4, 1)] |
| Funnel chart | funnel (meta1) |
| Egger's test | metabias (meta1, method="linreg") |
| The sensitivity analysis | metainf (meta1, pooled = "random") forest (metainf (meta1, pooled = "random"), xlim=c(0, 0.2)) |
| Subgroup analysis | meta1<-metaprop(event, n, study, data=rate, sm="PLN", incr=0.5, allincr=TRUE, addincr=FALSE, title="", byvar= subgroup title, print.byvar=TRUE) |
| Meta-regression analysis | metareg (meta1, ~covariate title) |

**Supplementary Material 3.** Included studies and quality scores**.**

|  | **Reference ID** | **No. tested** | **No. positive** | **Prevalence** | **Random sampling or not** | **Detection method clearly or not** | **Sampled method detailly or not** | **Sample time clearly or not** | **Four or more risk factors or not** | **Score** |
| --- | --- | --- | --- | --- | --- | --- | --- | --- | --- | --- |
| 1 | Xu et al. (2020) | 232 | 26 | 0.112069 | N | Y | N | Y | Y | 3 |
| 2 | Deng et al. (2020) | 901 | 20 | 0.022198 | N | Y | N | Y | Y | 3 |
| 3 | Guo et al. (2020) | 302 | 135 | 0.44702 | Y | Y | N | Y | Y | 3 |
| 4 | Chang et al. (2021) | 1234 | 89 | 0.072123 | N | Y | N | Y | Y | 3 |
| 5 | Lee et al. (2019) | 207 | 14 | 0.067633 | N | Y | N | Y | Y | 3 |
| 6 | Wang et al. (2017) | 81 | 6 | 0.074074 | N | Y | N | Y | Y | 3 |
| 7 | Chen et al. (2018) | 149 | 29 | 0.194631 | N | Y | N | Y | Y | 3 |
| 8 | Zhang (2016) | 173 | 24 | 0.138728 | N | Y | N | Y | y | 3 |
| 9 | Luo et al (2015) | 248 | 28 | 0.112903 | N | Y | N | Y | Y | 3 |
| 10 | Quan and Liu (2014) | 184 | 27 | 0.146739 | N | Y | N | Y | Y | 3 |
| 11 | Lv and Zhang (2014) | 252 | 58 | 0.230159 | N | Y | N | Y | Y | 3 |
| 12 | Fernández-Aguilar et al. (2016) | 133 | 79 | 0.593985 | N | Y | N | Y | Y | 3 |
| 13 | Kaveh et al. (2016) | 128 | 26 | 0.203125 | N | Y | N | Y | Y | 3 |
| 14 | Li et al. (2013) | 80 | 71 | 0.888 | N | Y | N | Y | Y | 3 |
| 15 | Decaro et al. (2017) | 1005 | 17 | 0.016915 | N | Y | N | Y | Y | 3 |
| 16 | Yao et al. (2019) | 145 | 19 | 0.131034 | N | Y | N | Y | Y | 3 |
| 17 | Song et al. (2019) | 382 | 101 | 0.264398 | N | Y | N | Y | Y | 3 |
| 18 | Wang and Man (2020) | 690 | 114 | 0.165217 | N | Y | N | Y | Y | 3 |
| 19 | Wei et al. (2020) | 640 | 256 | 0.4 | N | Y | N | Y | Y | 3 |
| 20 | Dehkordi (2011) | 992 | 203 | 0.204637 | N | Y | N | Y | Y | 3 |
| 21 | Weng (2015) | 4327 | 18 | 0.00416 | N | Y | N | Y | Y | 3 |
| 22 | Caffarena et al. (2021) | 480 | 6 | 0.0125 | N | Y | Y | Y | Y | 4 |
| 23 | Zhang et al. (2020) | 535 | 185 | 0.345794 | N | Y | Y | Y | Y | 4 |
| 24 | Long (2019) | 76 | 21 | 0.276316 | N | Y | Y | Y | Y | 4 |
| 25 | Li (2019) | 305 | 17 | 0.055738 | N | Y | Y | Y | Y | 4 |
| 26 | Wang (2017) | 232 | 35 | 0.150862 | N | Y | Y | Y | Y | 4 |
| 27 | Li (2019) | 109 | 87 | 0.798165 | N | Y | N | Y | Y | 4 |
| 28 | Yan et al. (2019) | 138 | 62 | 0.449275 | N | Y | N | Y | Y | 4 |
| 29 | Sun and Qin (2019) | 114 | 28 | 0.245614 | N | Y | Y | Y | Y | 4 |
| 30 | Han et al. (2018) | 143 | 87 | 0.608392 | N | Y | Y | Y | Y | 4 |
| 31 | Paixão et al. (2018) | 305 | 110 | 0.360656 | Y | Y | N | Y | Y | 4 |
| 32 | Wang et al. (2020) | 1160 | 14 | 0.012069 | Y | Y | N | Y | Y | 4 |
| 33 | Wang (2020) | 200 | 35 | 0.175 | Y | Y | N | Y | Y | 4 |
| 34 | Agah et al.(2019) | 1075 | 2 | 0.00186 | N | Y | Y | Y | Y | 4 |
| 35 | Viana et al. (2017) | 400 | 157 | 0.3925 | N | Y | Y | Y | Y | 4 |
| 36 | Zhang et al. (2019) | 1286 | 4 | 0.00311 | N | Y | Y | Y | Y | 4 |
| 37 | Freitas et al. (2021) | 6465 | 115 | 0.017788 | N | Y | Y | Y | Y | 4 |
| 38 | Wang et al. (2012) | 1434 | 23 | 0.016039 | Y | Y | Y | Y | Y | 5 |
| 39 | Yang et al. (2019) | 74 | 28 | 0.378378 | N | Y | N | Y | Y | 5 |
| 40 | Liu et al. (2017) | 346 | 317 | 0.916185 | Y | Y | Y | Y | Y | 5 |
| 41 | Lv et al. (2013) | 464 | 2 | 0.00431 | Y | Y | Y | Y | Y | 5 |
| 42 | Zhang et al. (2015) | 920 | 34 | 0.036957 | Y | Y | Y | Y | Y | 5 |
| 43 | Stephenson et al. (2017) | 7544 | 24 | 0.003181 | Y | Y | Y | Y | Y | 5 |
| 44 | Yan et.al (2018) | 400 | 146 | 0.365 | N | N | N | Y | Y | 2 |
| 45 | Wang. (2014) | 3503 | 1979 | 0.56494 | N | Y | N | Y | Y | 3 |
| 46 | Sha et al.(2014) | 842 | 178 | 0.2114 | N | Y | N | Y | Y | 3 |
| 47 | Huang. (2016) | 667 | 228 | 0.34183 | N | Y | N | Y | Y | 3 |
| 48 | Chen et al. (2017) | 190 | 88 | 0.46316 | N | Y | N | Y | Y | 3 |
| 49 | Wang. (2017) | 786 | 749 | 0.95293 | N | Y | N | Y | Y | 3 |
| 50 | Lu et al. (2018) | 150 | 74 | 0.49333 | N | Y | N | Y | Y | 3 |
| 51 | Sun et al. (2019) | 900 | 129 | 0.14333 | N | Y | N | Y | Y | 3 |
| 52 | Zhao et al. (2020) | 210 | 165 | 0.78571 | N | Y | N | Y | Y | 3 |
| 53 | Fu et al. (2012) | 1650 | 795 | 0.48182 | N | Y | N | Y | Y | 3 |
| 54 | Liu. (2016) | 522 | 333 | 0.63793 | N | Y | N | Y | Y | 3 |
| 55 | Wang et al. (2016) | 191 | 124 | 0.64921 | N | Y | N | Y | Y | 3 |
| 56 | Zhao (2016) | 326 | 294 | 0.90184 | N | Y | N | Y | Y | 3 |
| 57 | Olmo et al. (2019) | 520 | 30 | 0.05769 | N | Y | N | Y | Y | 3 |
| 58 | Zhu (2020) | 2358 | 1958 | 0.83036 | N | Y | N | Y | Y | 3 |
| 59 | Kumar et al. (2018) | 500 | 66 | 0.132 | N | Y | N | Y | Y | 3 |
| 60 | Zhong et al. (2016) | 604 | 113 | 0.18709 | N | Y | Y | Y | Y | 3 |
| 61 | Chen et al. （2016 | 1332 | 452 | 0.33934 | N | Y | N | Y | Y | 3 |
| 62 | Liu and Sun . (2014) | 192 | 39 | 0.20313 | N | Y | N | Y | Y | 3 |
| 63 | Dong et al. (2014) | 492 | 244 | 0.49593 | N | Y | N | Y | Y | 3 |
| 64 | He et al. (2014) | 1070 | 474 | 0.44299 | N | Y | N | Y | Y | 3 |
| 65 | Shang et al. (2013) | 1198 | 282 | 0.23539 | N | Y | N | Y | Y | 3 |
| 66 | Rodríguez-Prieto V et al. (2016) | 180 | 82 | 0.45556 | N | Y | N | Y | Y | 3 |
| 67 | Ma et al. (2016) | 1584 | 595 | 0.37563 | N | Y | N | Y | Y | 3 |
| 68 | Qu et al. (2016) | 1637 | 1013 | 0.61881 | N | Y | N | Y | Y | 3 |
| 69 | Shen et al. (2011) | 571 | 43 | 0.07531 | N | Y | N | Y | Y | 3 |
| 70 | Maya et al. (2016) | 390 | 298 | 0.7641 | N | Y | N | Y | Y | 3 |
| 71 | Liu (2017) | 559 | 202 | 0.36136 | N | Y | N | Y | Y | 3 |
| 72 | Cheng et.al (2017) | 920 | 448 | 0.48696 | N | Y | N | Y | Y | 3 |
| 73 | Luo et al. (2018) | 897 | 179 | 0.19955 | N | Y | N | Y | Y | 3 |
| 74 | Zhu (2017) | 559 | 202 | 0.36136 | N | Y | N | Y | Y | 3 |
| 75 | Gan et al. (2020) | 455 | 36 | 0.07912 | N | Y | N | Y | Y | 3 |
| 76 | Lin. (2015) | 741 | 491 | 0.66262 | N | Y | Y | Y | Y | 4 |
| 77 | Bi et al. (2020) | 1601 | 969 | 0.60525 | N | Y | Y | Y | Y | 4 |
| 78 | Cao et al. (2016) | 86 | 17 | 0.19767 | N | Y | Y | Y | Y | 4 |
| 79 | Liu et al. (2012) | 549 | 343 | 0.62477 | Y | Y | N | Y | Y | 4 |
| 80 | Li et al. (2013) | 665 | 472 | 0.70977 | Y | Y | N | Y | Y | 4 |
| 81 | Wang et al. (2021) | 456 | 75 | 0.16447 | N | Y | Y | Y | Y | 4 |
| 82 | Han et al. (2010) | 252 | 54 | 0.21429 | N | Y | Y | Y | Y | 4 |
| 83 | Luo (2017) | 920 | 448 | 0.48696 | Y | Y | N | Y | Y | 4 |
| 84 | Chen (2016) | 204 | 65 | 0.31863 | Y | Y | N | Y | Y | 4 |
| 85 | Hu and Gu (2016) | 917 | 450 | 0.49073 | Y | Y | N | Y | Y | 4 |
| 86 | Cheng et al. (2016) | 420 | 221 | 0.52619 | Y | Y | N | Y | Y | 4 |
| 87 | Liu et al. (2019) | 325 | 243 | 0.7477 | Y | Y | N | Y | Y | 4 |
| 88 | Zhao. (2020) | 389 | 179 | 0.46015 | Y | Y | N | Y | Y | 4 |
| 89 | Liu et al. (2020) | 792 | 518 | 0.65404 | Y | Y | N | Y | Y | 4 |
| 90 | Kang et al. (2013) | 546 | 14 | 0.02564 | Y | Y | N | Y | Y | 4 |
| 91 | Lei et al. (2013) | 188 | 170 | 0.90426 | Y | Y | N | Y | Y | 4 |
| 92 | Zhang et al. (2013) | 460 | 292 | 0.63478 | Y | Y | N | Y | Y | 4 |
| 93 | Yue et al. (2014) | 266 | 102 | 0.38346 | Y | Y | N | Y | Y | 4 |
| 94 | Maa et al. (2020) | 9016 | 2378 | 0.26375 | Y | Y | N | Y | Y | 4 |
| 95 | Demil et al. (2021) | 339 | 91 | 0.26844 | Y | Y | Y | Y | Y | 5 |
| 96 | Noaman and Nabinejad (2020) | 216 | 114 | 0.52778 | Y | Y | Y | Y | Y | 5 |
| 97 | Li (2018) | 516 | 267 | 0.51744 | Y | Y | Y | Y | Y | 5 |
| 98 | Segura-Correa JC et al. (2016) | 385 | 184 | 0.47792 | Y | Y | Y | Y | Y | 5 |
| 99 | Zhang et al. (2018) | 460 | 237 | 0.51522 | Y | Y | Y | Y | Y | 5 |
| 100 | Liu (2018) | 597 | 296 | 0.49581 | Y | Y | Y | Y | Y | 5 |
| 101 | Xie et al. (2015) | 385 | 374 | 0.97143 | Y | Y | Y | Y | Y | 5 |
| 102 | Chen et al. (2016) | 238 | 163 | 0.68487 | Y | Y | Y | Y | Y | 5 |
| 103 | Uddin MA et al. (2017) | 94 | 48 | 0.51064 | Y | Y | Y | Y | Y | 5 |
| 104 | Li et al. (2020) | 440 | 34 | 0.07727 | Y | Y | Y | Y | Y | 5 |
| 105 | Liu . (2020) | 1446 | 1244 | 0.8603 | Y | Y | Y | Y | Y | 5 |
| 106 | Erfani et al. (2019) | 562 | 161 | 0.286 | Y | Y | Y | Y | Y | 5 |
| 107 | Yuan et al. (2013) | 244 | 144 | 0.59016 | Y | Y | Y | Y | Y | 5 |
| 108 | Liu (2014) | 566 | 247 | 0.4364 | Y | Y | Y | Y | Y | 5 |
| 109 | Yao (2015) | 793 | 587 | 0.74023 | Y | Y | Y | Y | Y | 5 |
| 110 | Singh et al. (2017) | 466 | 71 | 0.15236 | Y | Y | Y | Y | Y | 5 |
| 111 | Katochn et al. (2017) | 132 | 2 | 0.01515 | Y | Y | Y | Y | Y | 5 |
| 112 | Chowdhury et al. (2015) | 94 | 48 | 0.51064 | N | N | Y | Y | Y | 3 |
| 113 | Asnake et al. (2020) | 225 | 19 | 0.08444 | Y | Y | Y | Y | Y | 5 |
| 114 | Tadesse et al. (2019) | 420 | 217 | 0.51667 | Y | Y | N | Y | Y | 4 |
| 115 | Daves et al. (2016) | 407 | 135 | 0.33170 | Y | Y | Y | Y | Y | 5 |
| 116 | Manandhar et al. (2018) | 350 | 9 | 0.02571 | Y | Y | N | Y | Y | 4 |
| 117 | Guidoum et al. (2020) | 234 | 138 | 0.58974 | Y | Y | Y | Y | Y | 5 |
| 118 | Berg et al. (2019) | 364 | 195 | 0.53571 | N | Y | Y | Y | Y | 4 |
| 119 | Olmo et al. (2018) | 151 | 12 | 0.07947 | N | Y | Y | Y | Y | 4 |
| 120 | Nugroho et al. (2020) | 77 | 9 | 0.11688 | N | Y | N | Y | Y | 3 |
| 121 | Mishran et al. (2014) | 1049 | 1 | 0.00095 | Y | Y | Y | Y | Y | 5 |
| 122 | Gangil et al. (2020) | 55 | 0 | 0 | N | Y | N | Y | Y | 3 |
| 123 | Alam et al. (2016) | 644 | 21 | 0.03261 | N | N | N | Y | Y | 2 |
| 124 | Yitagesu et al. (2021) | 1379 | 449 | 0.32560 | Y | Y | Y | Y | Y | 5 |
| 125 | Asmare et al. (2018) | 563 | 185 | 0.32860 | N | Y | Y | Y | Y | 4 |
| 126 | Maya et al. (2020) | 2546 | 23 | 0.00903 | N | Y | N | Y | Y | 2 |
| 127 | Ryu and Choi (2019) | 635 | 35 | 0.05512 | Y | Y | Y | Y | Y | 5 |
| 128 | Kim et al. (2019) | 3050 | 21 | 0.00689 | N | Y | Y | Y | Y | 4 |

Y*: Yes; N*: No.

**Supplementary Material 4.** Prevalence of bovine viral diarrhea virus in the world from 2010 to 2021


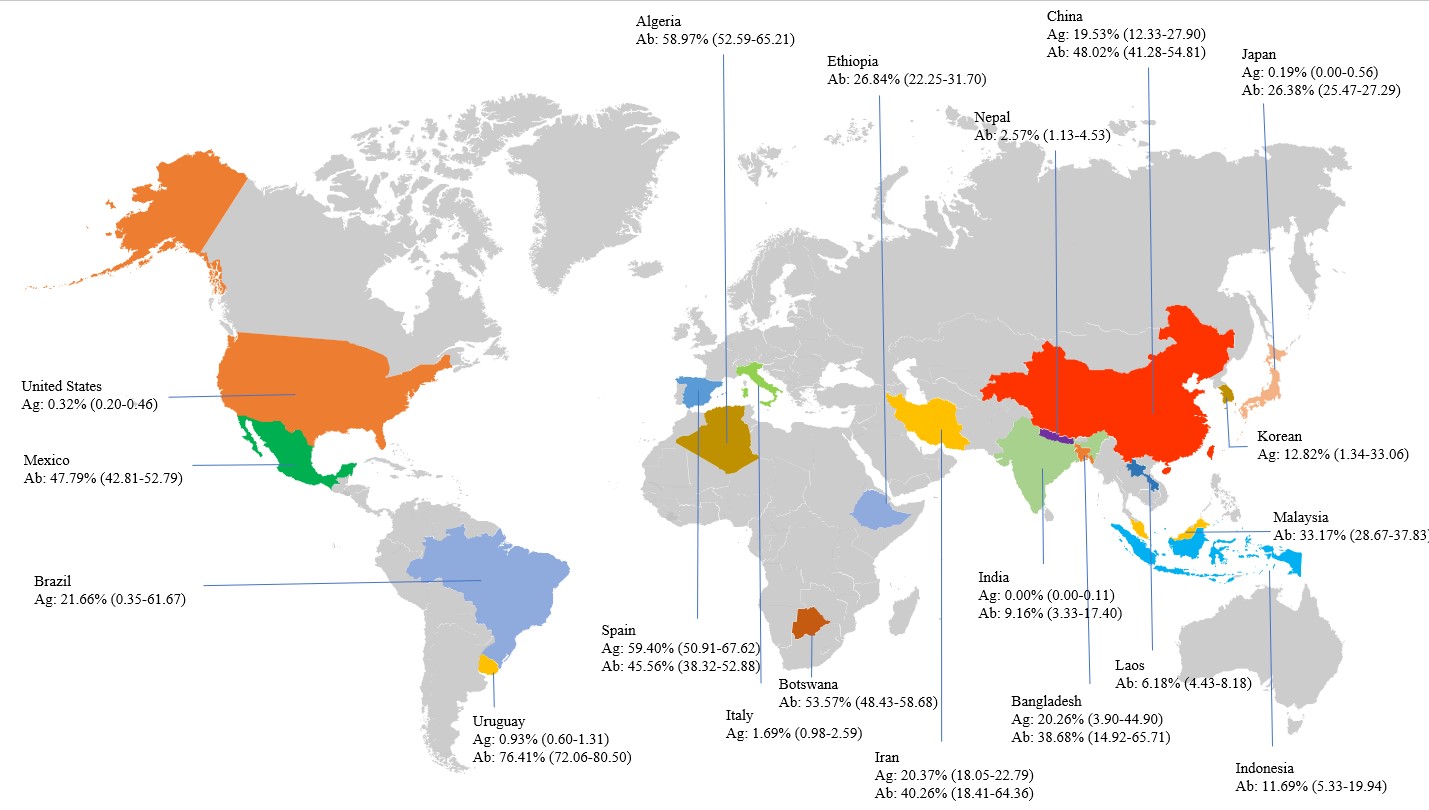


**References**

Wang W. Serosurvey of Major Bovine Resporitary Viruses and Identification of BVDV Isolates and Vaccine Development[D]. [Beijing]: Chinese Academy of Agricultural Sciences (2014).

Sha JM, Ka ZC, Wang ZS. Serological investigation of three viral diarrhea diseases in cattle herds in Huangnan Prefecture, Qinghai Province[J]. *Anim Husb Vet Med* (2014) 46 (10): 94-96. Arrived from: https://kns.cnki.net/kcms/detail/detail.aspx?FileName=XMYS201410027&DbName=CJFQ2014

Lin XY. The Infection Status Investigation of Five Major Diseases in Large-scale Farms in Parts of Shandong Province[D]. [Taian (SD)]: Shandong Agricultural University (2015).

Huang ZL. The Serosurvey of Major Diseases in Cattle Farms in Parts of Guangxi Province[D]. [Nanning (GX)]: Guangxi Univeresity (2016).

Chen M, Liu P, Cheng ZL, Liu SD. Seroepidemiological survey of common infectious diseases in large-scale dairy farms in Shandong Province[J]. *China Cattle Science* (2017) 43 (04): 74-77. Arrived from: https://kns.cnki.net/kcms/detail/detail.aspx?FileName=BULL201704024&DbName=CJFQ2017

Wang QQ. Epidemiological Investigation and Analysis of Bovine Viral Diarrhea in Partial Scale Cattle Farms of the South Xinjiang. [Aral (XJ)]: Tarim University (2017).

Lu CM, Yan ZG, Wang J. Serological investigation and Study on cow viral diarrhea in Jiading and Chongming areas of Shanghai[J]. *Veterinary Guide* (2018) (09): 73-75. Arrived from: https://kns.cnki.net/kcms/detail/detail.aspx?FileName=DWBJ201809042&DbName=CJFQ2018

Sun WM, Zhu J, Shen LH, Cao XY, Jin YC, Xu K, et al. Serological investigation of bovine viral diarrhea in Songjiang District, Shanghai[J]. *Shanghai Journal of Animal Husbandry and Veterinary Medicine* (2019) (02): 50+52. doi:10.14170/j.cnki.cn31-1278/s.2019.02.016

Zhao XL, Niu JQ, Ciren YJ, Ciren YZ, Suolang ZG, Wen DX, et al. Epidemiological Survey of Yak Diarrhea Virus BVDV, BCV and BRV[J]. *Plateau agriculture* (2020) 4 (03): 298-302. doi: 10.19707/j.cnki.jpa.2020.03.013

Bi YY, Song LL, Xue Y, Li JB, Li CH, Jia AQ, et al. Detection of Serum Antibodies against Major Pathogens Leading to BRDC in Large-scale Dairy Farms in Four Provinces，Northern China[J]. *China Animal Quarantine* (2020) 37 (12): 9-13. Arrived from: https://kns.cnki.net/kcms/detail/detail.aspx?FileName=ZGDW202012003&DbName=CJFQ2020

Fu CX, Jin XJ, Zheng RF, Guo F, Han L, Li J, et al. Serological investigation of three viral diarrhea diseases in large-scale dairy farms in Beijing[J]. *Progress in Veterinary Medicine* (2012) 33 (05): 85-88. doi:10.16437/ j.cnki.1007-5038.2012.05.018

Xu CQ, Hou HJ, Jiang X, Fu XB, Li LA, Ai X, et al. Isolation, Characterization and Molecular epidemiology of Bovine Viral Diarrhea Virus in Tianjin from 2018 to 2019[J]. *J Infect Dis* (2020) 28 (04): 24-28. Arrived from: https://kns.cnki.net/kcms/detail/detail.aspx?FileName=ZSJB202004004&DbName=CJFQ2020

Liu JQ. Investigation on bovine viral diarrhea in some large-scale dairy farms in Xinjiang[J]. *Modern Animal Husbandry* (2016) (02): 6-7. doi: 10.14070/j.cnki.15-1150.2016.02.00

Deng ML, Chen N, Guidarini C, Xu ZH, Zhang JJ, Cai LJ, et al. Prevalence and genetic diversity of bovine viral diarrhea virus in dairy herds of China. *Vet Microbiol* (2020) 242:108565. doi: 10.1016/j.vetmic.2019.108565

Guo T, Zhang JH, Chen XD, Wei X, Wu CX, Cui Q, et al. Investigation of viral pathogens in cattle with bovine respiratory disease complex in Inner Mongolia, China. *Microb Pathog* (2021) 153:104594. doi: 10.1016/j.micpath.2020.104594

Demil E, Fentie T, Vidal G, Jackson W, Lane J, Mekonnen SA, et al. Prevalence of bovine viral diarrhea virus antibodies and risk factors in dairy cattle in Gondar city, Northwest Ethiopia. *Prev Vet Med* (2021) 191:105363. doi: 10.1016/j.prevetmed.2021.105363

Caffarena RD, Casaux ML, Schild CO, Fraga M, Castells M, Colina R, et al. Causes of neonatal calf diarrhea and mortality in pasture-based dairy herds in Uruguay: a farm-matched case-control study. *Braz J Microbiol* (2021) 52(2):977-988. doi: 10.1007/s42770-021-00440-3

Chang LL, Qi YP, Liu D, Du Q, Zhao XM, Tong DW. Molecular detection and genotyping of bovine viral diarrhea virus in Western China. *BMC Vet Res* (2021) 17(1):66. doi: 10.1186/s12917-021-02747-7

Zhang L, Wang JL, Sun YY, Yang HJ, Zhang YF, Jiang RX, et al. Pathogenic detection of viruses related to calf diarrhea in dairy farms in Shandong Province during 2017 to 2018. *Chin J Anim Health Insp* (2020) 37 (6), 5. doi: CNKI:SUN:ZGDW.0.2020-06-005

Wang XL, Wang YM, Zhang YL, Wu YW, Li ZX, Zhang W, et al. Serological investigation of bovine viral diarrhea in some regions of Ningxia[J] *Chin J Anim Health Insp* (2016) 33 (2): 17-19. doi: 10.3969/j.issn.1005-944x.2016.02.008

Zhao SY. The isolation and Fabrication of E0 genetic prokaryptic expression vector of bovine viral diarrhea virus isolated from Ningxia[D]. [Yinchuan (NX)]: Ningxia University (2016). doi: 10.7666/d.y3109188

Long MC. Epidemiological investigation of bovine viral diarrhea virus[J]. *Today Animal Husbandry and Veterinary Medicine* (2019) 35 (5): 15. doi: 10.3969/j.issn.1673-4092.2019.05.011

Cao ST, Guo YN, Lei YY, Bai XN, Ma Y, Xu YT, et al. Serological investigation on the causes of abortion in large-scale dairy farms in Wuzhong area of Ningxia[J]. *Progress in Veterinary Medicine* (2016) 37 (4): 115-119. doi: 10.3969/j.issn.1007-5038.2016.04.025

Wang JT, Sang XB, Shi QW, Diao CX, Zhuang YL, et al. Serological investigation of bovine viral diarrhea antigens in large-scale dairy farms in Heilongjiang Province[J]. *Heilongjiang Anim Sci Vet Med* (2012) (24): 91-92. Arrived from: https://kns.cnki.net/kcms/detail/detail.aspx?FileName=ZSYZ202105003&DbName=CJFQ2021

Li JK, Liu MY, Han ZQ, Zhang KR, Shen MY, Bao SK, et al. Seroprevalence of Bovine Viral Diarrhea infection in yaks (Bos grunniens) in some counties of Qinghai- -Tibetan plateau, China. Chinese society of zootechnics and veterinary science - Chinese Veterinary Clinical Congress (2012). Arrived from: https://kns.cnki.net/kcms/detail/detail.aspx?FileName=ZGXJ201208001047&DbName=CPFD2012

Li DL, Zhao JY, Shen JL, Shi CJ, Cao J. Risk assessment and epidemic analysis of infectious rhinotracheitis and bovine viral diarrhea in dairy farms in Beijing[J]. *China dairy cattle* (2013) (11): 31-33. doi: 10.3969/j.issn.1004-4264.2013.11.009

Lee SH, Kim HY, Choi EW, Kim D. Causative agents and epidemiology of diarrhea in Korean native calves. *J Vet Sci* (2019) 20(6):e64. doi: 10.4142/jvs.2019.20.e64

Olmo L, Reichel MP, Nampanya S, Khounsy S, Wahl LC, Clark BA, et al. Risk factors for Neospora caninum, bovine viral diarrhoea virus, and Leptospira interrogans serovar Hardjo infection in smallholder cattle and buffalo in Lao PDR. *PLoS One* (2019) 14(8):e0220335. doi: 10.1371/journal.pone.0220335

Noaman V, Nabinejad AR. Seroprevalence and risk factors assessment of the three main infectious agents associated with abortion in dairy cattle in Isfahan province, Iran. *Trop Anim Health Prod* (2020) 52(4):2001-2009. doi: 10.1007/s11250-020-02207-8

Li ZY. Investigation on the main pathogens of calf diarrhea in Henan Province and analysis of two pathogens. [Zhengzhou (HA)]: Henan Agricultural University (2019).

Wang HR. The Investigation of Major Pathogens of the Calf Diarrhea and the Analysis of Biological Characteristics of E. coli in 13 provinces, China. [Beijing]: Chinese Academy of Agricultural Sciences (2017).

Wang MC, Yue H, Tang C, Yang ZL. Detection and genetic evolution of diarrhea-related viruses in Chongqing beef cattle[J]. *China animal husbandry and veterinary* (2017) 44 (09): 2731-2738. doi: 10.16431/j.cnki.1671-7236.2017.09.027

Chen XN, Xiao M, Ruan WQ, Qin SN, Yue H, Tang C, et al. Molecular epidemiological investigation and isolation of bovine viral diarrhea virus in yak in Sichuan-Tibet plateau region[J]. *Chin J Anim Vet Sci* (2018) 49 (03): 606-613. Arrived from: https://kns.cnki.net/kcms/detail/detail.aspx?FileName=XMSY201803018&DbName=CJFQ2018

Li J. Investigation and analysis of yak diarrhea in Qinghai [J]. *Today Animal Husbandry and Veterinary Medicine* (2019) 35 (11): 23. doi: 10.3969/j.issn.1673-4092.2019.11.016

Yan ZY, Lv BL, La J, Hai CX, Li WY. Detection and analysis of five viral pathogens associated with diarrhea in yaks in Huangzhong County, Qinghai Province[J]. *Anim Husb Vet Med* (2019) 51 (01): 88-92. Arrived from: https://kns.cnki.net/kcms/detail/detail.aspx?FileName=XMYS201901018&DbName=DKFX2019

Yang XL, Li ZQ, Luo YZ, Lu GH. Etiological investigation and analysis of viral diarrhea of Yaks in Xining, Qinghai[J]. *Chin J Vet Med* (2019) 55 (08): 8-12. Arrived from: https://d.wanfangdata.com.cn/periodical/ChlQZXJpb2RpY2FsQ0hJTmV3UzIwMjIxMjIyEg96Z3N5enoyMDE5MDgwMDIaCDFkNGN2YWpw

Sun L, Qin J. Epidemiological investigation on the pathogen of calf diarrhea in Bazhou, Xinjiang[J]. *Heilongjiang Anim Sci Vet Med* (2019) (24): 3. Arrived from: https://kns.cnki.net/kcms/detail/detail.aspx?FileName=HLJX201924018&DbName=DKFX2019

Li JK, Li K, Han ZQ, Zhang H, Wang XQ, Luo HQ, et al. Serological Survey of Bovine Viral Diarrhoea Virus among Yaks (Bos poephagus grunniens) in Hongyuan of Sichuan, China. *Pakistan Journal of Zoology* (2018) 50 (4): 1557-1559. doi: 10.17582/journal.pjz/2018.50.4.sc7

Wang LP, Jin XD, Bi JL, Su YS, Yang C, Li JL, Li KX, Yin GF. Seroepidemiological investigation of bovine viral diarrhea mucosal disease in Yunnan Province[J]. *China cattle science* (2021) 47 (01): 19-22. Arrived from: https://kns.cnki.net/kcms/detail/detail.aspx?FileName=BULL202101006&DbName=CJFQ2021

Zhu GY. Epidemiological investigation of BVDV in a large cattle farm in South Xinjiang. [Aral (XJ)]: Tarim University (2020).

Han DG, Ryu JH, Park J, Choi KS. Identification of a new bovine viral diarrhea virus subtype in the Republic of Korea. *BMC Vet Res* (2018) 14(1):233. doi: 10.1186/s12917-018-1555-4

Kumar SK, Palanivel KM, Sukumar K, Ronald BSM, Selvaraju G, Ponnudurai G. Herd-level risk factors for bovine viral diarrhea infection in cattle of Tamil Nadu. *Trop Anim Health Prod* (2018) 50(4):793-799. doi: 10.1007/s11250-017-1497-z

Zhang K. Investigation of calves viral diarrhea related pathogen of large-scale dairy farm in northern XinJiang region. [Shihezi (XJ)]: Shihezi University (2016).

<https://kns.cnki.net/KCMS/detail/detail.aspx?dbname=CMFD201701&filename=1016778661.nh>

Zhong YM, Zhang JF, Zhang J, Wang M, Su J, Zhang JY, et al. Monitoring and purification of breeding cattle disease in Heilongjiang Province[J]. *Chung-kuo Hsu Mu Shou I* (2016) (11): 17-18. Arrived from: https://kns.cnki.net/kcms/detail/detail.aspx?FileName=XMKX201611011&DbName=CJFQ2016

Chen R, Fan XZ, Zhu YY, Zou XQ, Xu L, Zhang QY, et al. Prevalence Study and Phylogenetic Analysis of Bovine Viral Diarrhea Virus in Free-Roaming Beef Cattle in Western China[J]. *Scientia Agricultural Sinica* (2016) 49 (13): 2634-2641. Arrived from: https://kns.cnki.net/kcms/detail/detail.aspx?FileName=ZNYK201613019&DbName=CJFQ2016

Luo YJ, Li J, Su GC, Qu YG, Cao SZ, Li Y. Investigation on infection of major viral reproductive disorders in dairy farms [J]. *China Anim Health Insp* (2015) 32 (06): 15-17. Arrived from: https://kns.cnki.net/kcms/detail/detail.aspx?FileName=ZGDW201506005&DbName=CJFQ2015

Liu P, Sun JW. Serological investigation on viral diarrhea and mucosal disease of dairy cows in Qian County, Shaanxi Province[J] *Farm Technology PC Digest magazine* (2014) 000 (006): 140-140. Arrived from: https://d.wanfangdata.com.cn/periodical/ChlQZXJpb2RpY2FsQ0hJTmV3UzIwMjIxMjIyEhFuamtqLXh4azIwMTQwNjEzNhoIa3l3aTZscDY%3D

Dong YS, Liu XQ, Li HR, Wang Y, Luo ZQ, Tang WS. Serological investigation of bovine viral diarrhea / mucosal disease in Qinghai Province[J]. *Heilongjiang Anim Sci Vet Med* (2014) (08): 66-67. doi: 10.13881/j.cnki.hljxmsy.2014.0815

Quan YC, Liu HS. Investigation on the infection of three bovine viral diarrhea pathogens in some areas of Qinghai Province[J]. *Chung-kuo Hsu Mu Shou I* (2014) 41 (05): 220-223. Arrived from: https://d.wanfangdata.com.cn/periodical/ChlQZXJpb2RpY2FsQ0hJTmV3UzIwMjIxMjIyEg96Z3htc3kyMDE0MDUwNDgaCGF0NDN0N3l5

LV JJ, Zhang LQ. Epidemiological investigation of viral diarrhea and infectious rhinotracheitis in Qinghai Yaks[J]. *Acta Ecologiae Animalis Domastici* (2014) 35 (04): 59-63. Arrived from: https://kns.cnki.net/kcms/detail/detail.aspx?FileName=JCST201404014&DbName=CJFQ2014

He ML, Zhang HR, Wang Y, Wang YX, Wang YW, Tang C. Serological investigation on three viral diarrhea diseases of Yaks in Northwest Sichuan[J]. *Chung-kuo Hsu Mu Shou I* (2014) 41 (03): 248-251. Arrived from: https://kns.cnki.net/kcms/detail/detail.aspx?FileName=GWXK201403069&DbName=CJFQ2014

Shang YP, Liu H, Zhang HL, Gao MC, Zhang WL, Wang JW. Serological investigation of bovine viral diarrhea-mucosal disease on scale dairy farms in the Northeast China[J]. *Chung-kuo Yu Fang Shou I Hsueh Pao* (2013) 35 (07): 559-561. Arrived from: https://kns.cnki.net/kcms/detail/detail.aspx?FileName=ZGXQ201307012&DbName=CJFQ2013

Han ZH, Quan H, He XL, Wei KF, Erdenizabu. Serological survey of viral diarrhea / mucosal disease and infectious rhinotracheitis in yaks[J]. *Chung-kuo Tung Wu Ch'uan Jan Ping Hsueh Pao* (2010) 18 (06): 56-59. Arrived from: https://kns.cnki.net/kcms/detail/detail.aspx?FileName=ZSJB201006013&DbName=CJFQ2010

Rodríguez-Prieto V, Kukielka D, Rivera-Arroyo B, Martínez-López B, de las Heras AI, et al. Evidence of shared bovine viral diarrhea infections between red deer and extensively raised cattle in south-central Spain. *BMC Vet Res* (2016) 12:11. doi: 10.1186/s12917-015-0630-3

Segura-Correa JC, Zapata-Campos CC, Jasso-Obregón JO, Martinez-Burnes J, López-Zavala R. Seroprevalence and risk factors associated with bovine herpesvirus 1 and bovine viral diarrhea virus in North-Eastern Mexico. *Open Vet J* (2016) 6(2):143-9. doi: 10.4314/ovj.v6i2.12

Ma JG, Cong W, Zhang FH, Feng SY, Zhou DH, Wang YM, et al. Seroprevalence and risk factors of bovine viral diarrhoea virus (BVDV) infection in yaks (Bos grunniens) in northwest China. *Trop Anim Health Prod* (2016) 48(8):1747-1750. doi: 10.1007/s11250-016-1118-2

Fernández-Aguilar X, López-Olvera JR, Marco I, Rosell R, Colom-Cadena A, Soto-Heras S, et al. Pestivirus in alpine wild ruminants and sympatric livestock from the Cantabrian Mountains, Spain. *Vet Rec* (2016) 178(23):586. doi: 10.1136/vr.103577

Zhang XJ, Yang Y, Lin H, Zhu GQ. Epidemiological survey of bovine viral diarrhea and infectious bovine rhinotracheitis in dairy herds of Jiangsu province[J]. *Chinese Journal of veterinary science* (2018) 38 (01): 69-76. doi: 10.16303/j.cnki.1005-4545.2018.01.10

Liu SQ. Serological investigation and analysis of cow viral diarrhea in some areas of Xinyang City, Henan Province. *China dairy* (2018) (11), 54-56. doi: 10.16172/j.cnki.114768.2018.11.016

Liu XP, Song K, Gao YW, Fu XZ, Wang ZS, Wu T. Serological investigation on major infectious diseases of beef cattle and crossbred beef cattle in Wujiaqu City, Xinjiang[J]. *China Cattle Science* (2017) 43 (06): 73-75.

Luo RB. Tibet bovine viral diarrhea disease epidemiology investigation and virus isolation and identification of sequence analysis[D]. [Lhasa (Tibet)]: Tibet University (2017).

Qu P, Zhao BL, Hu DM, Shi H, Cao DS, Song XH. Investigation on the prevalence of bovine viral diarrhea in Western China[J]. *Heilongjiang Anim Sci Vet Med* (2016) (06): 111-113. doi: 10.13881/j.cnki.hljxmsy.2016.0487

Xie CF, Yu RS, Li Z, Zhang RH, Si FS, Dong SJ. Epidemiological investigation on infectious rhinotracheitis and bovine viral diarrhea in large-scale dairy farms [J]. *China Dairy Cattle* (2016) (04): 38-41. doi: 10.19305/j.cnki.11-3009/s.2016.04.0010

Chen XL. Epidemiological survey on cattle disease in Sanming city[J]. *Fujian journal of animal husbandry and veterinary* (2016) 38 (05): 1-3. Arrived from: https://kns.cnki.net/kcms/detail/detail.aspx?FileName=FJCY201605001&DbName=CJFQ2016

Chen FM, Cheng GM, Ma AX, Hu SL. Serological investigation of bvd-md, IBR and TB in dairy cows in Weifang City and surrounding areas[J]. *Heilongjiang Anim Sci Vet Med* (2016) (18): 114-117. doi: 10.13881/j.cnki.hljxmsy.2016.1593

Hu RL, Gu JT. Epidemiological investigation of bovine viral diarrhea in large-scale dairy farms in Suzhou[J]. *Chung-kuo Hsu Mu Shou I* (2016) 32 (02): 115-116. Arrived from: https://kns.cnki.net/kcms/detail/detail.aspx?FileName=ZSYZ202105003&DbName=CJFQ2021

Cheng ZL, Liu P, Liu SD. Epidemiological study on three common epidemics in large-scale cattle farms in Shandong[J]. *China Cattle Science* (2016) 42 (01): 44-48.

Kaveh A, Merat E, Samani S, Danandeh S, Soltan Nezhad S. Infectious Causes of Bovine Abortion in Qazvin Province, Iran. *Arch Razi Inst* (2017) 72(4):225-230. doi: 10.22092/ari.2017.113299

Uddin MA, Ahasan ASML, Islam K, Islam MZ, Mahmood A, Islam A, et al. Seroprevalence of bovine viral diarrhea virus in crossbred dairy cattle in Bangladesh. *Vet World* (2017) 10(8):906-913. doi: 10.14202/vetworld.2017.906-913

Paixão SF, Fritzen JTT, Alfieri AF, Alfieri AA. Virus neutralization technique as a tool to evaluate the virological profile for bovine viral diarrhea virus infection in dairy water buffalo (Bubalus bubalis) herds. *Trop Anim Health Prod* (2018) 50(4):911-914. doi: 10.1007/s11250-017-1503-5

Wang HM, Li XH, Zhang HJ, Zhang LG, Zhang XL, Yin LL, et al. Report on detection results of bovine viral diarrhea in Weichang County, Hebei Province in 2017[J]. Veterinary Orientation (2020) (20): 2.

Liu ZY, Liu ZY, Li ZJ, Guo Li, Zhang JL. Investigation of BVDV, IBRV and BRSV infection in some cattle farms of Jilin Province[J]. *Anim Husb Vet Med* (2019) 51 (12): 101-105. Arrived from: https://kns.cnki.net/kcms/detail/detail.aspx?FileName=XMYS201912020&DbName=DKFX2019

Wang TL. Investigation of BVD infection and isolation and identification of epidemic strains in an area of Henan[D]. [Aral (XJ)]: Tarim University (2020).

Li BL, Tao J, Huang Z, Zhan T, Ma YL, Yu HF, et al. Serological investigation of bovine viral diarrhea in a dairy farm[J]. *Shanghai Journal of Animal Husbandry and Veterinary Medicine* (2020) (04): 40 + 42. doi: 10.14170/j.cnki.cn31-1278/s.2020.04.015

Zhao N. Serological investigation and Study on cow viral diarrhea disease in Pingjibao area of Ningxia [J]. *Gansu animal and veterinary sciences* (2020) 50 (01): 51-53. doi: 10.15979/j.cnki.cn62-1064/s.2020.01.020

Liu GS. Epidemiological investigation and prevention of IBR and BVD of dairy cows in Ningxia[D]. [Yinchuan (NX)]: Ningxia University (2020).

Liu ZY. Epidemiological investigation and gene sequence analysis of the main strain of BVDV of beef cattle in some areas of Jilin Province[D]. [Changchun (JL)]: Jilin Agricultural University (2020).

Shen YL, Cai JS, Li J, Hu GW, Wang XR. Serological investigation of bovine viral diarrhea mucosal disease in Yushu District of Qinghai Province[J]. *Chin J Anim Health Insp* (2011) 28 (10): 52. Arrived from: https://kns.cnki.net/kcms/detail/detail.aspx?FileName=ZGDW201110029&DbName=CJFQ2011

Li J, Li Y, Fan WX, Yuan LG, Qi YY, Pu JW, et al. Serological investigation of five epidemic diseases in some large-scale dairy farms in Xinjiang[J]. *Progress in Veterinary Medicine* (2013) 000 (011): 24-27. doi: 10.3969/j.issn.1007-5038.2013.11.006

Erfani AM, Bakhshesh M, Fallah MH, Hashemi M. Seroprevalence and risk factors associated with bovine viral diarrhea virus and bovine herpes virus-1 in Zanjan Province, Iran. *Trop Anim Health Prod* (2019) 51(2):313-319. doi: 10.1007/s11250-018-1687-3

Agah MA, Notsu K, El-Khaiat HM, Arikawa G, Kubo M, Mitoma S, et al. Slaughterhouse survey for detection of bovine viral diarrhea infection among beef cattle in Kyushu, Japan. *J Vet Med Sci* (2019) 81(10):1450-1454. doi: 10.1292/jvms.19-0045

Maya L, Puentes R, Reolón E, Acuña P, Riet F, Rivero R, et al. Molecular diversity of bovine viral diarrhea virus in uruguay. *Arch Virol* (2016) 161(3):529-35. doi: 10.1007/s00705-015-2688-4

Viana RB, Monteiro BM, Souza DC. Sensitivity and specificity of indirect ELISA for the detection of antibody titers against BVDV from beef cattle raised in Pará State[J]. *Semina Ciências Agrárias* (2017) 38(5):3049-3058. doi: info:doi/10.5433/1679-0359.2017v38n5p3049

Decaro N, Lucente MS, Lanave G, Gargano P, Larocca V, Losurdo M, et al. Evidence for Circulation of Bovine Viral Diarrhoea Virus Type 2c in Ruminants in Southern Italy. *Transbound Emerg Dis* (2017) 64(6):1935-1944. doi: 10.1111/tbed.12592

Liu Q. Serological investigation of Qinghai yak viral diarrhea virus[J]. *Contemporary livestock and poultry breeding* (2017) (03): 10-11. doi: 10.14070/j.cnki.15-1150.2017.03.008

Cheng SL, Wang G, Yixi CM, Luo RB, Zhou HB, Gong G, et al. Detection of serum antibody against viral diarrhea in Tibetan yaks [J]. *Hubei Journal of Animal and Veterinary Science* (2017) 38 (05): 5-6. doi: 10.16733/j.cnki.issn1007-273x.2017.05.001

Luo RB, Chen JC, Qu J, Cheng SL, Shen MY, Luo XL, et al. Serological investigation of viral diarrhea in Tibet yak[J]. *Journal of Plateau Agriculture* (2018) 2 (03): 261-265. doi: 10.19707/j.cnki.jpa.2018.03.007

Zhu L. Serological investigation of viral diarrhea in Qinghai yak[J]. *Contemporary livestock and poultry breeding* (2018) (07): 62-63. doi: 10.14070/j.cnki.15-1150.2018.07.057

Yan XL, He YC, Li XR, Li S. Serological investigation of bovine viral diarrhea mucosal disease and infectious rhinotracheitis in Zhangye City[J]. *China Cattle Science* (2018) 44 (03): 48-49.

Zhang SX, He MR, Yu HJ, He BN, Zhao SQ, Wang L, et al. Detection of Bovine viral diarrhea virus using qRT-PCR combined with double-antibody sandwich ELISA in a large-scale cattle farm in Heilongjiang Province[J]. *Heilongjiang Anim Sci Vet Med* (2019) (22): 72-75. doi: 10.13881/j.cnki.hljxmsy.2019.01.0308

Yao ZL, Fu HQ, Cui PF, Zong JL. Molecular epidemiology of bovine viral diarrhea virus and identification of a BVDV -2 isolate in regional area of Jiangsu and Zhejiang provinces[J]. *Journal of Yangzhou University (agricultural and Life Science Edition)* (2019) 40 (01): 40-46 + 102. doi: 10.16872/ j.cnki.1671-4652.2019.01.007

Song WB, Ma CS, Guo JM, Ma LT, Zhang XY. Etiological investigation and analysis of yak infected with bovine viral diarrhea virus and bovine enterovirus in Haibei Prefecture, Qinghai Province from 2016 to 2017[J]. *Heilongjiang Anim Sci Vet Med* (2019) (02): 88-90. doi: 10.13881/j.cnki.hljxmsy.2018.03.0120

Wang H, Man HY. Epidemiological investigation and analysis of bovine viral diarrhea virus in Liangzhou District, Wuwei City, Gansu Province[J]. *China dairy cattle* (2020) (12): 26-29. doi: 10.19305/j.cnki.11-3009/s.2020.12.007

Gan FB, Luo RB, Zhaxi CR, Baima SZ, Zhaxi WJ, Suolang SZ. Serological detection and analysis of antibody to yak viral diarrhea in bange County, Tibet [J]. *Gansu animal and veterinary sciences* (2020) 50 (10): 68-70. doi: 10.15979/j.cnki.cn62-1064/s.2020.10.020

Wei Q, Qu YG, Chang JS, Gu SY, Wu YY, Yu HJ, et al. Molecular epidemiological investigation of bovine viral diarrhea in some areas of Xinjiang[J]. *Anim Husb Vet Med* (2020) 52 (12): 105-109.

Safarpoor Dehkordi F. Prevalence study of Bovine viral diarrhea virus by evaluation of antigen capture ELISA and RT-PCR assay in Bovine, Ovine, Caprine, Buffalo and Camel aborted fetuses in Iran. *AMB Express* (2011) 1(1):32. doi: 10.1186/2191-0855-1-32

Kang XD, Xie XL, Wu SR, Ma C, Bai LJ. Serological investigation of viral diarrhea mucosal disease in dairy cows in Ningxia[J]. *Heilongjiang Anim Sci Vet Med* (2013) (04): 90-91. doi: 10.13881/j.cnki.hljxmsy.2013.04.036

Lei CH, Guo FL, Wei L, Shu Z, Lv CH, Huang YB, et al. Serological survey of bovine viral diarrhea-mucosal disease[J]. *Chin J Vet Med* (2013) 49 (04): 18-19. Arrived from: https://kns.cnki.net/kcms/detail/detail.aspx?FileName=ZGXJ201208001049&DbName=CPFD2012

Lv JJ, Gao LY, Shang HZ. Serological investigation of bovine viral diarrhea mucosal disease in Haixi District of Qinghai Province[J]. *Chinese Qinghai Journal of Animal and Veterinary Sciences* (2013) 43 (06): 35. Arrived from: https://kns.cnki.net/kcms/detail/detail.aspx?FileName=QXSZ201306024&DbName=CJFQ2013

Zhang GW, Li Y, Hao WF, Shi XT. Epidemiological investigation of cow viral diarrhea in Taiyuan area[J]. *China dairy* (2013) (07): 39-42. Arrived from: https://kns.cnki.net/kcms/detail/detail.aspx?FileName=ZGNN201307012&DbName=CJFQ2013

Yuan XJ, Zhang Y, Li NZ, Li CM, Yang BF, Wang ZL, et al. Serological investigation of six major bovine infectious diseases in some areas of Southwest China [C] / / National Conference on bovine disease control and industrial development Huazhong Agricultural University; Laboratory of disease control function of national modern agriculture (beef / yak) industrial technology system (2013).

Liu MY. Seroprevalence of bovine viral diarrhea infection in yaks and BVDV vaccine, swine fever vaccine immune effects on yaks. [Wuhan (HB)]: Huazhong Agricultural University (2014).

Yue RC, Cheng ZL, Li N, Liu SD. Serological survey and analysis of bovine common infectioussome cattle farms in Shandong province[J]. *China Anim Health Insp* (2014) 31 (06): 58-61.

Weng XG. Epidemiologic survey of bovine viral diarrhea in Beijing region, Study of IFN- α/β response in persistently infected cattle and immunomodulatory effects of forsythoside A[D]. China Agricultural University (2015).

Zhang X, Huang KH, Zhang KC. Serological investigation of two viral diseases in dairy cows in Shanghai[J]. *China diary* (2015) (14): 54-59. doi: 10.3969/j.issn.1004-4264.2015.14.015

Yao W. Serologic study on bovine viral diarrhea in diary cattle from scale dairy farms in Liaoning[J]. *Modern journal of animal husbandry and veterinary medicine* (2015) (04): 36-40. doi: 10.3969/j.issn.1672-9692.2015.04.007

Stephenson MK, Palomares RA, White BJ, Engelken TJ, Brock KV. Prevalence of bovine viral diarrhea virus (BVDV) persistently infected calves in auction markets from the southeastern United States; association between body weight and BVDV-positive diagnosis. *The Professional Animal Scientist* (2017) 33(4): 426-431. Arrived from: <https://doi.org/10.15232/pas.2017-01619>

Akagami M, Seki S, Kashima Y, Yamashita K, Oya S, Fujii Y, et al. Risk factors associated with the within-farm transmission of bovine viral diarrhea virus and the incidence of persistently infected cattle on dairy farms from Ibaraki prefecture of Japan. *Res Vet Sci* (2020) 129:187-192. doi: 10.1016/j.rvsc.2020.02.001

Freitas BB, Correa A, Valotto AA, Marcom NN, Paulino LR, Brum JS, et al. Prevalence of bovines persistently infected with bovine viral diarrhea virus (BVDV) in dairy cattle herds in Paraná State, Brazil[J]. *Pesquisa Veterinária Brasileira* (2021) 41(9). doi: 10.1590/1678-5150-pvb-6622

Singh V, Mishra N, Kalaiyarasu S, Khetan RK, Hemadri D, Singh RK, et al. First report on serological evidence of bovine viral diarrhea virus (BVDV) infection in farmed and free ranging mithuns (Bos frontalis). *Trop Anim Health Prod* (2017) 49(6):1149-1156. doi: 10.1007/s11250-017-1310-z

Katoch S, Dohru S, Sharma M, Vashist V, Chahota R, Dhar P, et al. Seroprevalence of viral and bacterial diseases among the bovines in Himachal Pradesh, India. *Vet World* (2017) 10(12):1421-1426. doi: 10.14202/vetworld.2017.1421-1426

Chowdhury MMR, Afrin F, Saha SS, Jhontu S, Asgar MA. Prevalence and haematological parameters for bovine viral diarrhoea (BVD) in South Bengal areas in Bangladesh[J]. *Bangladesh Veterinarian* (2015) 32(2): 48-54. doi: https://doi.org/10.3329/bvet.v32i2.30610

Asnake P, Lemma A, Tesfaye A., Gizaw D, Guta S, Dima C, et al. Seroprevalence of Bovine Viral Diarrhea Virus (BVDV) and Its Associated Risk Factors in Dairy Cattle in and Around Assela Town, South East Ethiopia[J]. *Research Article* (2020). doi: https://doi.org/10.21203/rs.3.rs-128860/v1

Tadesse T, Deneke Y, Deresa B. Seroprevalence of bovine viral diarrhea virus and its potential risk factors in dairy cattle of Jimma town, southwestern Ethiopia[J]. *J Dairy, Vet Anim Res* (2019) 8(1): 11-17. doi:10.15406/jdvar.2019.08.00235

Daves L, Yimer N, Arshad SS, Sarsai K, Omar MA, Yusoff R, et al. Seroprevalence of bovine viral diarrhea virus (BVDV) infection and associated risk factors in cattle in Selangor, Malaysia[J]. V*et. Med. Open J* (2016) 1: 22-28. doi: 10.17140/VMOJ-1-105

Manandhar S, Yadav GP, Singh DK. Epidemiological survey of bovine viral diarrhea in dairy cattle in Nepal[J]. *OIE bulletin newsfeed (2018)*. doi: 10.20506/bull.2018.NF.2860

Guidoum KA, Benallou B, Pailler L, Espunyes J, Napp S, Cabezón O. Ruminant pestiviruses in North Africa[J]. *PREV VET MED* (2020) 184: 105156. doi: https://doi.org/10.1016/j.prevetmed.2020.105156

Berg M, Ramabu SS, Wensman JJ, Lysholm S. First-time detection of bovine viral diarrhoea virus, BVDV-1, in cattle in Botswana[J]. *Onderstepoort J Vet Res* (2019) 86(1): 1-7. doi: https://hdl.handle.net/10520/EJC-1fddc2c775

Olmo L, Dye MT, Reichel MP, Youngd JR, Nampanyaac S, Khounsyc S, et al. Investigation of infectious reproductive pathogens of large ruminants: Are neosporosis, brucellosis, leptospirosis and BVDV of relevance in Lao PDR?[J]. *Acta tropica* (2018) 177: 118-126. doi: https://doi.org/10.1016/j.actatropica.2017.10.007

Nugroho W, Reichel MP, Ruff N, Gazali AM, Sakke IS. Infection with Bovine Viral Diarrhea Virus in cattle in Southern Papua, Indonesia[J]. *Acta Tropica* (2020) 212: 105712. doi: https://doi.org/10.1016/j.actatropica.2020.105712

Mishra N, Rajukumar K, Pateriya A, Kumar M, Dubey P, Behera SP, et al. Identification and molecular characterization of novel and divergent HoBi-like pestiviruses from naturally infected cattle in India. *Vet Microbiol* (2014) 7;174(1-2):239-46. doi: 10.1016/j.vetmic.2014.09.017

Gangil R, Kaur G, Dwivedi P N. Detection of respiratory viral antigens in nasal swabs of bovine by sandwich ELISA[J]. *Indian J Anim Res* (2020) 54: 354-358. doi: 10.10.18805/ijar.B-3769

Alam MR, Afrin K, Dash AK, Bhowmik DK, Sen AB, Nath S. Incidence and Therapeutic Management of Viral Diseases in Cattle at Jaintapur, Sylhet, Bangladesh[J]. *J Adv Res* (2016) 3(6): 13-20. soi: http://s-o-i.org/1.15/ijarm-2016-3-6-3

Yitagesu E, Jackson W, Kebede N, Smith W, Fentie T. Prevalence of bovine abortion, calf mortality, and bovine viral diarrhea virus (BVDV) persistently infected calves among pastoral, peri-urban, and mixed-crop livestock farms in central and Northwest Ethiopia. *BMC Vet Res* 17, 87 (2021) doi: https://doi.org/10.1186/s12917-021-02798-w

Asmare K, Sibhat B, Ayelet G, Gebremedhin EZ, Lidete KA, Skjerve E. Serological evidence of Bovine herpesvirus-1, Bovine Viral Diarrhea virus and Schmallenberg virus infections in relation to reproductive disorders in dairy cattle in Ethiopia[J]. *Acta tropica* (2018) 178: 236-241. doi: https://doi.org/10.1016/j.actatropica.2017.12.005

Maya L, Macías-Rioseco M, Silveira C, Giannitti F, Castells M, Salvo M, et al. An extensive field study reveals the circulation of new genetic variants of subtype 1a of bovine viral diarrhea virus in Uruguay[J]. *Archives of virology* (2020) 165(1): 145-156. doi: https://doi.org/10.1007/s00705-019-04446-z

Ryu J H, Choi K S. Genetic analysis of bovine viral diarrhea virus in pre-weaned native Korean calves[J]. *Trop Anim Health Pro* (2019) 51(7): 2085-2090. doi: https://doi.org/10.1007/s11250-019-01882-6

Kim Y, Kim Y, Lee SY, Lee KK, Lee KH, Song JC, et al. Identification of Korean native cattle persistently infected with BVDV using Ear-notch method[J]. *Korean j vet serv* (2019) 42(2): 117-120. doi: https://doi.org/10.7853/kjvs.2019.42.2.117

**Table S1.** Estimates of antigen positivity rates for bovine viral diarrhea virus around the world.

| Province | No.  studies | Region | No.  tested | No.  positive | % Prevalence | % (95% CI) |
| --- | --- | --- | --- | --- | --- | --- |
| Brazil | 3 | South America | 7170 | 382 | 21.66% | 0.35-61.67 |
| China | 31 | Asia | 17413 | 1913 | 19.53% | 12.33-27.90 |
| Bangladesh | 3 | Asia | 2586 | 655 | 20.26% | 3.90-44.90 |
| Iran | 2 | Asia | 1120 | 229 | 20.37% | 18.05-22.79 |
| India | 2 | Asia | 1104 | 1 | 0.00% | 0.00-0.11 |
| Italy | 1 | Europe | 1005 | 17 | 1.69% | 0.98-2.59 |
| Japan | 1 | Asia | 1075 | 2 | 0.19% | 0.00-0.56 |
| Korean | 4 | Asia | 4035 | 157 | 12.82% | 1.34-33.06 |
| Spain | 1 | Europe | 133 | 79 | 59.40% | 50.91-67.62 |
| United States | 1 | North America | 7544 | 24 | 0.32% | 0.20-0.46 |
| Uruguay | 2 | South America | 3026 | 29 | 0.93% | 0.60-1.31 |
| Total | 51 |  | 36290 | 2753 | 15.74% | 11.35-20.68 |

**Table S2.** Estimates of antibody positivity rates for bovine viral diarrhea virus around the world.

| Province | No.  studies | Region | No.  tested | No.  positive | % Prevalence | % (95% CI) |
| --- | --- | --- | --- | --- | --- | --- |
| China | 56 | Asia | 40227 | 20278 | 48.02% | 41.28-54.81 |
| Iran | 2 | Asia | 778 | 275 | 40.26% | 18.41-64.36 |
| Bangladesh | 4 | Asia | 833 | 332 | 38.68% | 14.92-65.71 |
| Ethiopia | 1 | Africa | 339 | 91 | 26.84% | 22.25-31.70 |
| India | 3 | Asia | 1098 | 139 | 9.16% | 3.33-17.40 |
| Japan | 1 | Asia | 9016 | 2378 | 26.38% | 25.47-27.29 |
| Laos | 2 | Asia | 671 | 42 | 6.18% | 4.43-8.18 |
| Mexico | 1 | North America | 385 | 184 | 47.79% | 42.81-52.79 |
| Spain | 1 | Europe | 180 | 82 | 45.56% | 38.32-52.88 |
| Uruguay | 1 | South America | 390 | 298 | 76.41% | 72.06-80.50 |
| Algeria | 1 | Africa | 234 | 138 | 58.97% | 52.59-65.21 |
| Botswana | 1 | Africa | 364 | 195 | 53.57% | 48.43-58.68 |
| Indonesia | 1 | Asia | 77 | 9 | 11.69% | 5.33-19.94 |
| Malaysia | 1 | Asia | 407 | 135 | 33.17% | 28.67-37.83 |
| Nepal | 1 | Asia | 350 | 9 | 2.57% | 1.13-4.53 |
| Total | 77 |  | 55349 | 24585 | 42.77% | 37.01-48.63 |

**Table S3.** Egger’s for Publication Bia.

| slope | bias | se.bias | t | df | p-value |
| --- | --- | --- | --- | --- | --- |
| -0.006923068 | 15.436945074 | 2.536751953 | 6.0853 | 49 | 1.729e-07 |

**Table S4.** Egger’s for Publication Bia.

| slope | bias | se.bias | t | df | p-value |
| --- | --- | --- | --- | --- | --- |
| 0.72347436 | 0.06041154 | 3.46906707 | 0.017414 | 75 | 0.9862 |

**Figure S1.** **Trim and fill analysis (detection antigen)**

**Figure S2. Trim and fill analysis (detection antibody)**

**Figure S3. Funnel plot with pseudo 95% confidence limits intervals for the examination of publication bias of sampling years (detection antigen)**

**Figure S4. Funnel plot with pseudo 95% confidence limits intervals for the examination of publication bias of season (detection antigen)**

**Figure S5. Funnel plot with pseudo 95% confidence limits intervals for the examination of publication bias of detection method (detection antigen)**

**Figure S6. Funnel plot with pseudo 95% confidence limits intervals for the examination of publication bias of health condition (detection antigen)**

**Figure S7. Funnel plot with pseudo 95% confidence limits intervals for the examination of publication bias of age (detection antigen)**

**Figure S8. Funnel plot with pseudo 95% confidence limits intervals for the examination of publication bias of Breeding mode (detection antigen)**

**Figure S9. Funnel plot with pseudo 95% confidence limits intervals for the examination of publication bias of study quality (detection antigen)**

**Figure S10. Funnel plot with pseudo 95% confidence limits intervals for the examination of publication bias of region (detection antigen)**

**Figure S11. Funnel plot with pseudo 95% confidence limits intervals for the examination of publication bias of country (detection antigen)**

**Figure S12. Funnel plot with pseudo 95% confidence limits intervals for the examination of publication bias of breed (detection antigen)**

**Figure S13. Funnel plot with pseudo 95% confidence limits intervals for the examination of publication bias of sample (detection antigen)**

**Figure S14. Funnel plot with pseudo 95% confidence limits intervals for the examination of publication bias of sampling years (detection antibody)**

**Figure S15. Funnel plot with pseudo 95% confidence limits intervals for the examination of publication bias of region (detection antibody)**

**Figure S16. Funnel plot with pseudo 95% confidence limits intervals for the examination of publication bias of country (detection antibody)**

**Figure S17. Funnel plot with pseudo 95% confidence limits intervals for the examination of publication bias of season (detection antibody)**

**Figure S18. Funnel plot with pseudo 95% confidence limits intervals for the examination of publication bias of health condition (detection antibody)**

**Figure S19. Funnel plot with pseudo 95% confidence limits intervals for the examination of publication bias of age (detection antibody)**

**Figure S20. Funnel plot with pseudo 95% confidence limits intervals for the examination of publication bias of gender (detection antibody)**

**Figure S21. Funnel plot with pseudo 95% confidence limits intervals for the examination of publication bias of breeding mode (detection antibody)**

**Figure S22. Funnel plot with pseudo 95% confidence limits intervals for the examination of publication bias of study quality (detection antibody)**

**Figure S23. Funnel plot with pseudo 95% confidence limits intervals for the examination of publication bias of breed (detection antibody)**

**Figure S24. Funnel plot with pseudo 95% confidence limits intervals for the examination of publication bias of detection method (detection antibody)**
